# The lactate–HCAR1 axis in exercise psychiatry: a candidate mechanism linking bone marrow–brain immunity to inflammation-related depression

**DOI:** 10.3389/fpsyt.2026.1871773

**Published:** 2026-07-17

**Authors:** Chuankai Luan, Chuanping Lei, Min Liu

**Affiliations:** 1 School of Physical Education, Qufu Normal University, Qufu, China; 2 College of Chinese Language and Literature, Qufu Normal University, Qufu, China; 3 School of Physical Education, Shandong Sports University, Rizhao, China

**Keywords:** bone marrow-brain axis, exercise psychiatry, GPR81, HCAR1, inflammation-related depression, lactate, lactate-informed exercise prescription framework, microglia

## Abstract

Exercise is increasingly acknowledged as an effective intervention for depression, yet the biological mechanisms underlying its antidepressant effects remain incompletely defined. Conventional models have emphasized brain-centered pathways, including BDNF signaling, monoaminergic modulation, hypothalamic-pituitary-adrenal axis regulation, hippocampal neurogenesis, and synaptic plasticity. These mechanisms are important, but they may not fully explain depression phenotypes characterized by elevated inflammatory burden, fatigue, anhedonia, diminished motivation, psychomotor slowing, cognitive inefficiency, and altered peripheral immune activity. This review proposes that exercise-induced lactate should be considered not only as a metabolic substrate or byproduct, but also as a dynamic immunometabolic signal. Appropriately dosed exercise can generate a transient and recoverable lactate pulse that may be detected by immune, vascular, and brain-associated cells through hydroxycarboxylic acid receptor 1, also known as HCAR1 or GPR81. We propose that the lactate-HCAR1 axis could represent a candidate mechanism linking exercise metabolism to bone marrow myeloid regulation, peripheral inflammatory tone, brain vascular and border interfaces, glial responses, and inflammation-related depressive symptom dimensions. The framework integrates skeletal muscle metabolism, myelopoiesis, peripheral myeloid cells, blood-brain interfaces, microglia, astrocytes, and endothelial cells into a cross-organ model of exercise psychiatry. Importantly, the strength of evidence differs across this pathway: some components are supported by human exercise physiology or preclinical causal studies, whereas others remain inferential and require validation in patients with depression. We therefore frame lactate-informed exercise prescription as a testable translational hypothesis rather than a clinically established strategy. Future studies should examine lactate kinetics, inflammatory biomarkers, immune cell phenotypes, symptom dimensions, safety, recovery dynamics, and comparisons with conventional heart-rate-, VO_2_-, or perceived-exertion-based prescriptions. If validated, this framework may help identify which patients benefit from exercise, which biological responses are meaningful, and how exercise can be studied as a quantifiable immunometabolic intervention.

## 1 Introduction

Exercise has transitioned from an adjunctive component of depression treatment to a central focus in mechanistic psychiatry. Meta-analytic evidence from randomized trials supports exercise as an effective intervention for depression, with clinically meaningful effects observed across aerobic, resistance, and mind–body modalities (1). However, the biological rationale for this phenomenon has been less thoroughly articulated than the intervention itself. Exercise induces considerable physiological alterations; nonetheless, its antidepressant mechanisms are often described using a brain-centered framework: regulation of the hypothalamic-pituitary-adrenal axis, modulation of monoamines, synaptic plasticity, hippocampal neurogenesis, and brain-derived neurotrophic factor (BDNF) (2). Although these mechanisms are significant, they do not comprehensively elucidate the diverse reactions to exercise, profound fatigue, anhedonia, and psychomotor retardation observed in certain patients, nor the enduring associations between inflammatory and metabolic markers and depressive disorders (3–6).

A key limitation of conventional exercise psychiatry is the tendency to treat major depression as a biologically uniform disorder and exercise as a uniform behavioral intervention. This assumption is increasingly difficult to sustain. Major depression includes subgroups with different inflammatory, metabolic, neuroendocrine, and behavioral profiles (3, 4). In this review, we use the term inflammation-related depression as a heuristic and stratification-oriented concept rather than as a formally established diagnostic subtype. It refers to depressive presentations characterized by elevated peripheral inflammatory markers, immune-metabolic burden, and/or symptom dimensions commonly associated with inflammation, including fatigue, diminished motivation, anhedonia, psychomotor slowing, sleep disturbance, and cognitive inefficiency (5–7). These features suggest disturbances in energy allocation, immune-metabolic communication, corticostriatal reward processing, and body-brain signaling. Therefore, neural plasticity models alone are unlikely to provide a complete explanation for the antidepressant effects of exercise in this phenotype. This definition is intentionally operational. It may include patients with elevated C-reactive protein, IL-6, TNF-α, or broader immune-metabolic signatures; patients with obesity, insulin resistance, metabolic syndrome, poor sleep, or other inflammatory comorbidities; and patients whose depressive symptoms are dominated by fatigue, anhedonia, reduced motivation, psychomotor slowing, hypersomnia, or cognitive inefficiency. These categories are not mutually exclusive, and none should be treated as a definitive biomarker-defined subtype at present. Instead, they provide a clinically useful framework for patient stratification in future mechanistic trials.

According to this review, exercise-induced lactate ties the mood-enhancing effects of exercise to immunometabolic pathways involved in inflammation-related depression. Historically, lactate was misunderstood as simply a byproduct of anaerobic metabolism or a marker of fatigue, but recent studies have challenged this view. Lactate is consistently produced, moved, oxidized, and shared between tissues, serving as a metabolic substrate and a signaling molecule (8, 9). During exercise, lactate is not simply increased; it is produced as a dynamic pulse, with its amplitude, timing, and recovery shaped by exercise intensity, training status, mitochondrial efficiency, tissue transport, and metabolic condition (8, 9). This temporal framework is essential. An increase in lactate that is short-lived and reversible may signal adaptation, while ongoing lactate irregularities might imply metabolic stress, inflammation, or a decrease in oxidative capacity.

HCAR1, also called GPR81, is identified as hydroxycarboxylic acid receptor 1, giving this framework specificity at the receptor level. Originally, HCAR1 was known as a lactate-sensitive G-protein-coupled receptor playing a role in metabolic regulation; however, later studies have connected lactate-HCAR1 signaling with the modulation of innate immune responses, vascular adaptation, and neurological responses (10–12). Lactate suppresses inflammatory pathways in macrophage and inflammasome models via GPR81-dependent mechanisms (11). Lactate produced during exercise triggers cerebral VEGF and angiogenesis via HCAR1, suggesting that lactate can convert physical effort into brain vascular changes (12). These findings elevate lactate from a mere passive exercise biomarker to a potential immunometabolic signaling molecule.

This review introduces the lactate-HCAR1 immunometabolic gate as a conceptual framework rather than as an established hypothetical integrative pathway. The term refers to a distributed, receptor-mediated checkpoint through which exercise-induced lactate may influence immune-cell behavior, vascular signaling, and neuroinflammatory tone. This gate is unlikely to be located at a single anatomical site. Instead, it may operate across skeletal muscle, bone marrow, circulating myeloid cells, vascular endothelium, brain-border tissues, astrocytes, microglia, and neurons. The psychiatric relevance of this pathway is likely to depend not only on lactate concentration, but also on lactate kinetics, HCAR1 expression, inflammatory priming, metabolic status, tissue compartment, and recovery dynamics.

The bone marrow-brain immune axis is central to this model. Long-term stress can activate hematopoietic stem and progenitor cells, boost myeloid cell production, and cause circulating immune cells to develop inflammatory characteristics (5, 6, 13–15). Stress-induced mobilization and trafficking of monocytes can influence brain inflammation and behavior, suggesting a connection between peripheral immune programming and mood and motivation. The integrity of the blood-brain barrier (BBB) may be compromised by stress, which can also change neurovascular interfaces, allowing peripheral inflammatory signals to more easily access reward and stress circuits (16). Taken together, these findings suggest that inflammation-related depression may involve dysfunctional immune responses and immune-to-brain signaling, instead of being due to inherent brain abnormalities.

Recent preclinical evidence provides an important but still limited mechanistic anchor for this framework. In depression-relevant animal models, running exercise has been reported to alleviate stress-related bone marrow myeloid skewing and neuroinflammation through a lactate-HCAR1/GPR81-related pathway (17). This finding shifts attention from the brain parenchyma alone to hematopoietic and vascular systems that may regulate inflammatory pressure on the brain. However, this evidence remains primarily preclinical, and direct causal validation in patients with depression is lacking. Therefore, the present review treats exercise-induced lactate-HCAR1 signaling as a candidate mechanism that may contribute to, rather than fully explain, the antidepressant effects of exercise in inflammation-related depression.

This framework may refine the approach to prescribing and studying exercise. Present guidelines usually measure exercise by weekly duration, session frequency, or broad intensity categories. These variables are clinically useful but biologically coarse. If lactate-HCAR1 signaling is implicated in antidepressant effects, a biologically relevant dose may be ascertained by the lactate response, encompassing its elevation, peak, duration, clearance, and correlation with symptoms and immune markers. In clinical and sports physiology, exercise intensity is tailored using blood lactate measurements and lactate-threshold principles (18). The subsequent step is to determine whether analogous principles can guide precision exercise psychiatry, especially in patients exhibiting inflammatory biomarkers, fatigue, anhedonia, metabolic syndrome, or insufficient responses to standard antidepressants.

Consequently, we examine exercise lactate not merely as a metabolic byproduct, but as a potential signal connecting movement, immunity, and mood. We reinterpret lactate in the field of psychiatry as a fluctuating immunometabolic signal, moving away from its traditional role as a static sign of physical exertion. Secondly, we examine the lactate-HCAR1 signaling pathway to differentiate receptor-mediated signaling from lactate transport, oxidation, and lactylation. Third, We explore the connection between bone marrow and brain immunity in inflammation-related depression, focusing on myelopoiesis, peripheral myeloid cells, brain barriers, and the convergence of glial and vascular systems. Fourth, we examine the impact of exercise-induced lactate-HCAR1 signaling on immune function in models of depression. We suggest a translational model for exercise prescriptions guided by lactate, viewing exercise as a measurable metabolic stimulus instead of a vague lifestyle factor. The assertion is that lactate is not the sole mechanism through which exercise exerts antidepressant effects. Exercise leads to the secretion of myokines, catecholamines, cortisol, adipokines, temperature changes, vascular shear stress, mitochondrial adaptations, and behavioral reinforcement. The signaling of lactate through HCAR1 provides a clear and controllable entry into this vast network. This process might explain how a metabolic signal from the periphery can lead to mental health improvements in cases of inflammation-related depression.

## 2 Reconceptualizing lactate in psychiatry: from a metabolic byproduct to an immunometabolic gateway

Exercise is increasingly recognized not only as a behavioral suggestion, but also as a biologically active treatment for depression. Meta-analytic evidence from randomized trials supports the clinically significant antidepressant effects of structured exercise, with variations depending on modality, intensity, and patient characteristics (1). Mechanistically, the field has frequently elucidated these benefits using a primarily neurocentric vocabulary: increased BDNF (BDNF), hippocampal neuroplasticity, modulation of monoaminergic transmission, normalization of hypothalamic-pituitary-adrenal axis function, and adult neurogenesis (2, 19, 20). These mechanisms are significant, but they are insufficient. Their explanation for the heterogeneity of exercise responses, the predominance of fatigue, anhedonia, and psychomotor slowing in certain depressed patients, and the relationship between peripheral inflammatory biomarkers and depressive symptom dimensions and treatment outcomes is inadequate (3, 5, 6, 13, 14).

We propose that exercise-induced lactate serves as a conceptual link between these neural models and the more general immunometabolic biology of depression. Lactate should no longer be thought of as a byproduct of glycolysis or a biochemical indicator of exercise. It can be thought of as a dynamic, temporally regulated signal that transmits peripheral metabolic demand to immune, vascular, and neurological systems (8, 9, 21, 22). Within this framework, the critical event is not a long-term increase in lactate levels, but rather a brief exercise-induced lactate surge. A pulse can activate receptor-dependent programs, particularly those involving hydroxycarboxylic acid receptor 1, also known as HCAR1 or GPR81, linking skeletal muscle metabolism to immune regulation, cerebral vascular adaptation, and neuroinflammatory tone (10–12, 22–26).

This recontextualization is particularly important for inflammation-related depression. If a subset of depression is characterized by altered peripheral immunity, myeloid-cell function, cytokine signaling, and impaired body-brain communication, exercise should not be viewed solely as a catalyst for “brain plasticity. “ It should also be recognized as a systemic metabolic disruption that can alter the immune pathways through which the body communicates with the brain (3, 5, 13, 14, 17).

### 2.1 Limitations of brain-centric models on the antidepressant effects of exercise

The brain-centric model of exercise as an antidepressant has proven to be effective. Exercise has been linked to changes in neurotrophic signaling, hippocampal volume and function, monoaminergic neurotransmission, stress hormone regulation, and synaptic plasticity (2, 19, 20). BDNF is important because it links physical activity to neuronal survival, synaptic remodeling, and antidepressant-like plasticity (20). The lactate literature initially supported the neuroplasticity-focused perspective: exercise-induced lactate can cross or affect brain regions, improving learning and memory through SIRT1-mediated activation of hippocampal BDNF signaling (27).

This model has a limitation. Depression is not a single neuronal plasticity disorder; rather, it is a heterogeneous syndrome with immune, metabolic, and vascular pathways predominating in specific subgroups. Meta-analysis shows that individuals with major depression have higher levels of inflammatory cytokines, such as IL-6 and TNF-α, compared to healthy controls (3). Furthermore, inflammation appears to be associated with specific symptom dimensions rather than simply exacerbating overall depression severity. Neurovegetative and motivational symptoms, such as fatigue, sleep disturbances, anhedonia, and psychomotor retardation, have strong links to inflammatory biology (5, 13, 14).

This dimensional pattern is difficult to understand solely using BDNF, monoamines, or hippocampal neurogenesis. Inflammatory signaling can reduce dopamine synthesis, release, and availability, owing to altered dopamine availability and basal ganglia functionality, providing a plausible biological pathway to anhedonia, decreased motivation, and psychomotor retardation (6, 28–30). These characteristics are clinically important, but they occur at the intersection of peripheral immunity and central motivational circuitry. A model that begins and ends in the brain risks missing the upstream biology through which exercise may produce antidepressant effects in inflammation-related depression.

The constraint is not the inaccuracy of brain plasticity models, but rather their overly narrow scope. Exercise is a comprehensive bodily intervention. It has an impact on skeletal muscle metabolism, circulating metabolites, endocrine regulation, immune cell trafficking, vascular signaling, and brain function (2, 8, 9, 19, 21, 31–33). A comprehensive model should investigate how peripheral signals caused by exercise are transformed into central psychiatric effects. Lactate is a strong candidate for this signal because of its rapid rise during exercise, dynamic clearance during recovery, role as a metabolic substrate, and involvement in receptor-mediated signaling pathways with immune and vascular implications (8–12, 21–26).

### 2.2 Lactate acts as a dynamic signal rather than a static metabolite

Lactate has long been misunderstood as a metabolic byproduct or a biochemical marker of fatigue. Exercise physiology has disproved this viewpoint. Lactate is constantly produced and used in aerobic conditions, acting as a transportable carbon source between glycolytic and oxidative tissues during exercise (8, 21). According to the lactate shuttle concept, lactate is not a byproduct of impaired metabolism, but rather a circulating intermediate that reallocates energy substrates between the muscle, heart, liver, and brain (8, 21).

In psychiatry, the primary significance of exercise lactate is its dynamics. Exercise does not merely cause a high lactate level. The generated pulse varies in amplitude, duration, and tissue interpretation depending on exercise intensity, muscle recruitment, training status, mitochondrial function, monocarboxylate transporter capacity, and recovery kinetics (8, 9, 18, 21, 34–36). This temporal profile is important because an acute lactate surge may indicate adaptation, whereas chronically disrupted lactate metabolism may indicate metabolic stress, mitochondrial dysfunction, hypoxia, or inflammatory reprogramming (8, 9, 21).

As a result, the psychiatric significance of lactate cannot be determined solely based on its concentration. A single molecule can have different effects depending on timing, compartmentalization, and cellular context. Lactate may act as an exerkine-like signal during properly calibrated exercise, orchestrating adaptive responses in both cerebral and peripheral tissues (8, 9). Lactate has been shown in experimental studies to mediate exercise-induced hippocampal BDNF signaling and cognitive benefits (27). Exercise-induced lactate can independently activate HCAR1-dependent pathways, increasing cerebral VEGF expression and angiogenesis, implying that lactate aids in the conversion of peripheral exertion into structural adaptations in the brain (12).

This dynamic perspective has a direct impact on inflammation-related depression. A brief lactate surge after exercise may not only “nourish” the brain, but may also reset the inflammatory pathways that the body uses to affect the brain. Lactate is better understood as a time-dependent immunometabolic signal rather than a static metabolite. The relevant biological question is: how much lactate exposure—intensity, duration, tissue specificity, and receptor involvement—is required to shift immune and neuroinflammatory conditions into an antidepressant phenotype?

### 2.3 The significance of HCAR1/GPR81

HCAR1/GPR81 is essential because it provides receptor specificity for lactate signaling. Without HCAR1, the term “lactate signaling” can become mechanistically ambiguous, encompassing lactate transport via monocarboxylate transporters, mitochondrial oxidation, redox equilibrium, epigenetic lactylation, and extracellular receptor activation. HCAR1 restricts this domain. Lactate has been identified as a ligand that activates a specific G-protein-coupled receptor, which was initially studied in metabolic tissues but has since been linked to brain and immune biology (10, 22, 23, 37).

This distinction is important for exercise psychiatry. If lactate is viewed solely as a fuel, its role in depression is limited to energy provision. When lactate is viewed as a receptor ligand, it emerges as a potential messenger through which exercise can influence immune and vascular health. Activation of HCAR1 has been linked to anti-inflammatory effects in innate immune pathways. lactate–HCAR1 signaling in macrophages and monocytes can inhibit inflammatory responses linked to Toll-like receptors and inflammasomes, including pathways involving NF-κB, NLRP3, and IL-1β (11). This provides a mechanistic basis for perceiving lactate as both a metabolic indicator of exercise intensity and a modulator of inflammatory tone.

HCAR1 is relevant to brain-related compartments. Exercise-induced lactate has been shown to stimulate cerebral VEGF and angiogenesis via HCAR1, with receptor enrichment observed in cells associated with brain-supplying vessels and intracerebral microvessels (12). This discovery is conceptually significant because it shows that lactate can interact with a specific receptor at vascular interfaces near the brain. These interfaces are where peripheral inflammatory signals can be filtered, amplified, or transformed before reaching neural circuits.

As a result, we use the term lactate-HCAR1 gate to describe a receptor-mediated checkpoint that may regulate immune-to-brain communication via exercise-induced lactate. This gate is unlikely to form a single anatomical structure. Instead, it may function in several compartments, including bone marrow, circulating myeloid cells, vascular endothelium, brain border tissues, perivascular cells, and glia. The function may be determined by the timing of lactate exposure, HCAR1 expression, inflammatory priming, metabolic health, and exercise dosage. This model is more accurate than simply stating “lactate is an antidepressant” because it implies that lactate gains psychiatric significance when processed by immune and vascular systems that express HCAR1.

### 2.4 Operational definition of inflammation-related depression

Inflammation-related depression is used here as an operational phenotype rather than a formal diagnostic category. The term refers to depressive presentations in which peripheral inflammatory activity, immune-metabolic burden, or inflammation-associated symptom dimensions are likely to be clinically relevant. This may include patients with elevated CRP, IL-6, TNF-α, or other inflammatory markers; patients with obesity, insulin resistance, metabolic syndrome, sleep disturbance, or other immune-metabolic comorbidities; and patients whose symptoms are dominated by fatigue, anhedonia, diminished motivation, psychomotor slowing, hypersomnia, or cognitive inefficiency (3–6, 13, 14).

This phenotype is a useful test case for the lactate-HCAR1 framework because peripheral-to-central immune communication is more likely to contribute to depressive symptoms in these patients than in depression presentations without clear inflammatory or metabolic burden. Inflammation is associated with altered reward processing, reduced motivation, fatigue, and psychomotor slowing, partly through effects on corticostriatal circuitry and dopamine availability (6, 13, 14). These symptoms are not merely indicators of global depression severity; they may reflect a biologically coherent immune-metabolic dimension.

Exercise has clinically meaningful antidepressant effects (1), but its mechanisms and efficacy are unlikely to be identical across all patients. Patients with inflammatory, metabolic, or somatic-motivational profiles may respond to exercise partly because exercise can modify peripheral immune tone and reduce inflammatory signaling to the brain. The lactate-HCAR1 pathway provides one plausible explanation for this transition. Recent preclinical evidence links running exercise to reduced depression-related myeloid skewing in bone marrow and reduced neuroinflammation through the lactate-HCAR1/GPR81 axis (17). However, this evidence requires replication, cell-specific validation, and human translation.

We therefore propose the following testable hypothesis: transient and recoverable exercise-induced lactate pulses may activate HCAR1-dependent immunometabolic programs that contribute to altered bone marrow myeloid output, reduced peripheral inflammatory signaling, modulation of brain-vascular and glial interfaces, and improvement in inflammation-related symptom dimensions such as fatigue, anhedonia, and diminished motivation. This hypothesis does not replace BDNF, monoamine, neurogenesis, HPA-axis, myokine, or behavioral reinforcement models. Rather, it adds a cross-organ immune-metabolic layer in which skeletal muscle metabolism, immune-cell dynamics, vascular interfaces, and brain plasticity are connected by a time-sensitive lactate-HCAR1 signal.

The implication is not that lactate-guided exercise prescription is currently ready for routine clinical use. Instead, lactate kinetics should be studied as a measurable biological exposure that may help refine future mechanism-based exercise trials. Relevant parameters may include lactate rise, peak concentration, time above threshold, area under the curve, clearance rate, recovery profile, inflammatory biomarkers, immune-cell phenotypes, and symptom-dimensional outcomes. **Table 1** outlines Evidence hierarchy for the proposed lactate-HCAR1-bone marrow-brain immunity pathway.

**Table 1:** Evidence hierarchy for the proposed lactate-HCAR1-bone marrow-brain immunity pathway.

Proposed mechanistic step	Main supporting evidence	Model system	HCAR1 dependence directly tested?	Human translational evidence	Remaining uncertainty
Exercise induces a transient lactate pulse	Strong exercise physiology literature showing intensity-dependent lactate production and recovery kinetics	Human and animal exercise studies	Not applicable	Strong. Exercise-induced lactate kinetics can be measured during graded exercise testing and controlled training protocols	Lactate responses vary by age, sex, fitness, metabolic status, medication use, sleep, diet, exercise modality, and inflammatory burden
Lactate activates HCAR1/GPR81	Receptor biology identifying lactate as an endogenous ligand for HCAR1/GPR81	Cell-based, structural, and animal studies	Yes	Moderate. HCAR1 biology is conserved, but psychiatric target engagement in humans remains unclear	Cell-type-specific HCAR1 signaling in immune, vascular, and brain-border compartments remains incompletely defined
Lactate-HCAR1 modulates innate immune signaling	Studies showing reduced macrophage or inflammasome-related inflammatory responses through GPR81-dependent pathways	Cell-based and animal inflammatory models	Yes in some studies	Limited. Human immune-cell data in depression are sparse	Some lactate effects are HCAR1-independent and may reflect MCT transport, redox changes, metabolic substrate use, or lactate oxidation/lactylation
Exercise lactate-HCAR1 alters bone marrow myeloid output in depression-relevant models	Running exercise reported to reduce stress-related myeloid skewing and neuroinflammation through the lactate-HCAR1/GPR81 axis	Preclinical depression models	Yes in the key preclinical model, but further validation is needed	Low. Direct human bone marrow evidence in depression is lacking	Forced versus voluntary exercise paradigms, lactate kinetics, cell specificity, and causal relevance to symptoms require validation
Peripheral myeloid remodeling reduces inflammatory pressure on brain interfaces	Stress and depression models link myeloid cells, cytokines, endothelial activation, and neuroinflammation	Mainly animal stress models, with indirect human biomarker evidence	Mostly unresolved	Low-to-moderate. Human evidence is mainly based on inflammatory markers, leukocyte profiles, imaging, and clinical associations	Causality from peripheral myeloid remodeling to antidepressant response in humans remains unproven
Brain-border, endothelial, microglial, and astrocytic changes mediate symptom improvement	Literature links BBB, choroid plexus, meningeal interfaces, endothelial cells, microglia, and astrocytes to stress-related behavioral changes	Animal models; selected human imaging and post-mortem studies	Mostly unresolved	Low-to-moderate. Human evidence exists for neuroinflammatory and vascular abnormalities in depression, but not for the full lactate-HCAR1 pathway	Direct evidence that exercise-induced lactate acts through HCAR1 at these interfaces in human depression is absent
Lactate-informed exercise prescription improves inflammation-related depression	Hypothesis derived from exercise physiology, lactate biology, immunometabolism, and inflammation-related depression literature	Proposed clinical trial framework	Not yet tested	Very low. No validated clinical protocol currently shows superiority over heart-rate-, VO_2_-, or RPE-based prescription	Requires prospective trials measuring lactate kinetics, inflammatory biomarkers, immune-cell phenotypes, symptoms, safety, adherence, and comparative efficacy

This table stratifies the proposed pathway according to evidence strength. The complete lactate-HCAR1-bone marrow-brain immunity pathway should be interpreted as a testable integrative framework rather than as a uniformly established causal chain. Human evidence is strongest for exercise-induced lactate kinetics, but remains limited for HCAR1-dependent bone marrow, brain-border, glial, and antidepressant mechanisms in patients with depression.

## 3 Lactate-HCAR1 signaling apparatus: dosage, timing, cellular types, and compartments

The biological significance of lactate is inextricably linked to its kinetics. In exercise psychiatry, lactate should not be viewed as a binary variable—present or absent, high or low—but rather as a signal whose effects vary depending on dosage, timing, cellular target, and anatomical compartment. During physical activity, circulating lactate reflects the balance of production, transport, uptake, oxidation, and clearance, rather than just glycolytic production (8, 9, 18, 21, 34–36). This feature is critical for psychiatric translation: two people participating in the same 30-minute running session may have significantly different lactate exposures due to training status, muscle recruitment, mitochondrial capacity, monocarboxylate transporter expression, autonomic tone, medication use, metabolic health, and recovery kinetics (8, 9, 18, 21, 34–36, 38).

This section distinguishes three levels of lactate biology. Initially, exercise lactate kinetics define the stimulus’s temporal and dosage framework. The biology of the HCAR1/GPR81 receptor determines whether extracellular lactate is recognized as a receptor-mediated signal by immune, vascular, and neural cells. To avoid conceptual overextension, non-HCAR1 lactate pathways—such as monocarboxylate transporter-mediated shuttling, mitochondrial oxidation, and lactylation—must be distinguished from HCAR1 signaling. Collectively, these distinctions allow the lactate-HCAR1 axis to be described as a verifiable immunometabolic signaling apparatus rather than a generic “lactate mechanism”.

### 3.1 Understanding exercise lactate kinetics: pulse, threshold, and recovery

Exercise generates lactate in a dynamic manner. Blood lactate concentration during exercise is influenced by both production and elimination rates, which include muscle generation, trans-sarcolemmal transport, hepatic and renal gluconeogenesis, oxidation by active and inactive tissues, and post-exercise clearance (8, 18, 21, 36). Classic and contemporary lactate shuttle models demonstrate that the majority of lactate produced during prolonged exercise is eventually oxidized rather than accumulating as waste (8, 21). As a result, the lactate response to exercise is better described as a dynamic flux system than as a fixed concentration.

This distinction is not purely semantic. In exercise-based psychiatry, lactate kinetics define the metabolic “exposure” provided by a specific training session. The relevant parameters include lactate rise rate, peak concentration, time above threshold, clearance slope, and recovery profile. These parameters may determine whether lactate acts as an adaptive signal activating receptor-mediated signaling or as an indicator of excessive metabolic stress. Blood lactate analysis is widely used in clinical exercise testing and endurance training to determine exercise intensity, physiological adaptation, and prescription zones (18, 34–36). The current opportunity is to incorporate this rationale into psychiatry: exercise dosage should be determined not only by duration, heart rate, or perceived exertion, but also by lactate dynamics, which link peripheral exertion to immune and cerebral signaling.

#### 3.1.1 Lactate threshold: a biological dosage parameter

The lactate threshold is a potential dosing parameter for precision exercise psychiatry. Lactate levels do not increase linearly with workload during incremental exercise; instead, they follow a curvilinear pattern with breakpoints indicating shifts in the production-clearance balance (34–36). Despite their heterogeneity and occasional methodological disputes, lactate threshold concepts remain useful for determining personalized exercise intensities and prescribing training loads with greater precision than fixed percentages of maximal heart rate or maximal oxygen uptake (34, 35).

This is significant because psychiatric exercise trials frequently classify exercise as “moderate intensity” or “vigorous intensity”. These categories may obscure significant interindividual differences in metabolic exposure. A sedentary person with metabolic syndrome, a trained runner, and a patient taking medications that affect autonomic or metabolic function can all do 30 minutes of treadmill exercise; however, their lactate curves may differ significantly. One person may remain below the lactate threshold, another may experience a transient adaptive surge, and a third may accumulate lactate with a delayed clearance rate. If lactate-HCAR1 signaling is involved in antidepressant effects, these individuals did not receive the same biological intervention.

Lactate thresholds should be viewed as more than just a metric for athletic performance. It could indicate a biological dosing threshold that distinguishes low-lactate aerobic activity from high-lactate metabolic signaling. In the context of inflammation-related depression, this threshold may help determine the exercise intensity required to activate lactate-sensitive immune and vascular pathways without causing excessive physiological stress. This is especially significant because the optimal psychiatric dosage is unlikely to be “maximum lactate intake”. The goal may be to generate a reproducible, manageable lactate pulse, followed by effective recovery.

#### 3.1.2 Acute lactate spikes versus chronic metabolic strain

The review distinguishes between acute exercise-induced lactate surges and chronic lactate dysregulation. Acute lactate elevation during exercise is typically transient and associated with increased energy flux, mitochondrial oxidation, and adaptive signaling (8, 9, 12). In this context, lactate may act as an exerkine-like signal that coordinates tissue adaptation, including cerebral vascular responses, hippocampal plasticity, and immune modulation (8, 9, 11, 12, 24, 27). In contrast, persistently elevated or insufficiently cleared lactate in pathological conditions may indicate mitochondrial dysfunction, hypoxia, inflammatory glycolysis, or maladaptive metabolic reprogramming (39, 40).

This distinction is critical in psychiatry because lactate has historically had ambiguous connotations. Lactate infusion in panic biology can cause panic-like symptoms in predisposed individuals, implying that lactate may be perceived as aversive or interoceptively threatening under certain conditions (41). In exercise biology, transient lactate production is considered a normal adaptive response. These observations do not conflict. Lactate signaling is context dependent. The same molecule may promote adaptation when produced as a transient, predictable, exercise-associated pulse, but it may cause distress or pathology when exposed to chronic metabolic stress, compromised clearance, altered acid-base sensing, or increased interoceptive threat.

In inflammation-related depression, the therapeutic question is not simply whether lactate should be increased, but what pattern of lactate exposure is adaptive, tolerable, and recoverable. A transient exercise-associated lactate pulse may contribute to HCAR1-mediated immune or vascular signaling (11, 12, 24, 26), whereas sustained elevation, impaired clearance, acid-base disturbance, or non-exercise lactate exposure may reflect metabolic strain or may produce aversive interoceptive effects (39–41). A minimal clinical validation design would include: baseline inflammatory stratification using CRP, IL-6, TNF-α, and metabolic markers (3–6, 13, 14); graded exercise testing to determine fitness and lactate threshold (18, 34–36); serial lactate sampling before exercise, immediately after exercise, and during recovery.

Preclinical exercise paradigms also require careful interpretation. Forced treadmill running allows better control of speed, duration, and workload, but it can introduce stress-related confounds such as handling, aversive stimulation, fatigue, and activation of stress pathways (42, 43). Voluntary wheel running is less aversive and may better reflect spontaneous activity, but it provides weaker control over intensity, timing, inter-individual variation, and the actual lactate pulse generated during exercise (44). This distinction is important for lactate-HCAR1 research because a given rodent exercise paradigm may not produce a well-characterized lactate exposure. Human studies may have an advantage in this respect, because graded exercise testing, serial blood lactate sampling, lactate-threshold assessment, heart-rate monitoring, and perceived exertion can be combined to define individualized lactate kinetics (18, 34–36).

### 3.2 HCAR1/GPR81: receptor biology and related pathways

HCAR1, also known as GPR81, provides receptor specificity to extracellular lactate signaling. It was first identified as a lactate-sensitive G-protein-coupled receptor that inhibits lipolysis in adipocytes through Gi-mediated cAMP suppression (10, 45). Subsequent research has expanded its biological significance beyond adipose tissue, involving HCAR1 in cerebral function, vascular adaptation, inflammation, neurogenesis, and tissue responses to metabolic stress (12, 22, 37, 46, 47). Recent structural studies have elucidated the molecular mechanisms underlying HCAR1 ligand recognition and Gi coupling, supporting the claim that HCAR1 is a true lactate-sensing receptor rather than an imprecise pharmacological surrogate for lactate exposure (48).

The established HCAR1 signaling mechanism involves Gi/o coupling and a decrease in intracellular cAMP; however, this is not the whole story (22, 48). HCAR1 activation can trigger Gβγ-dependent pathways, ERK, PI3K-Akt signaling, and β-arrestin-associated responses, depending on the cell type and context (22, 46, 48). The context dependence is central to the proposed model. HCAR1 activation in adipocytes, endothelial cells, macrophages, neurons, and glia should not be assumed to produce the same biological outcomes. Lactate-HCAR1 signaling should be regarded as a cell-state-dependent decoding mechanism, as immune cells, vascular interfaces, and neural compartments can interpret identical extracellular lactate signals differently.

#### 3.2.1 HCAR1 in immune cells

HCAR1 is particularly important in this review because it links lactate to immune regulation. Lactate interacting with GPR81 can block innate inflammatory pathways, including the NF-κB activity triggered by Toll-like receptors, the activation of the NLRP3 inflammasome, and the production of IL-1β (11). Lactate in macrophages can decrease the production of pro-inflammatory cytokines and change inflammatory metabolism, although some anti-inflammatory effects of lactate might happen without involving GPR81, highlighting the importance of differentiating between receptor-mediated and metabolic mechanisms (11, 24, 25).

Bone marrow plays an additional important role. During inflammation, neutrophils in the bone marrow can release lactate, which engages with endothelial GPR81 to enhance neutrophil movement (25). The significance of this finding lies in its mechanistic role, highlighting lactate–HCAR1 signaling as a key point where metabolism, vascular endothelium, and myeloid cell trafficking converge. This points to a likely mechanism by which lactate from exercise affects cytokine production and the generation, release, and movement of myeloid cells, all impacting brain inflammation in inflammation-related depression.

The immunological interpretation of HCAR1 is thus more than just “anti-inflammatory”. Lactate-HCAR1 signaling affects the positioning, activation levels, and movement of immune cells. It might suppress excessive inflammatory signals in monocytes and macrophages and could control neutrophil movement in bone marrow endothelial niches; and its effects in chronic inflammation may differ depending on whether immune cells, stromal cells, or vascular gatekeepers express HCAR1 (11, 24–26). The lactate-HCAR1 gate model of exercise psychiatry relies heavily on HCAR1 in the immune and bone marrow areas.

#### 3.2.2 HCAR1 in cerebrally associated compartments

HCAR1 is distributed throughout the brain in compartments that are strategically placed to translate lactate signals into changes in neural excitability, blood flow, vascular plasticity, and barrier function. Initial research identified HCAR1 expression and function in the mammalian brain, including the neocortex and hippocampus, and proposed that lactate could act as a volume transmitter between neuronal activity, cerebral blood flow, and energy metabolism (37). Activation of HCAR1 suppresses neuronal network activity through Gα and Gβγ subunits, whereas lactate receptor activation decreases neuronal activity in both rodent and human brain tissues (46, 47).

Nonetheless, the most important compartments for inflammation-related depression may include more than just neurons. Lactate-driven exercise enhances VEGF expression and angiogenesis in the brain via HCAR1, with receptor accumulation noted in pial fibroblast-like cells close to vessels supplying the brain and within intracerebral microvessels (12). Lactate receptor and transporter immunoreactivity has been found in human neuropathological studies in astrocytes and choroid plexus epithelial cells, indicating the possibility of lactate-sensitive interfaces at glial and cerebrospinal fluid barrier compartments (49). These findings imply that HCAR1 might function at the interface between the circulatory system and the brain, covering vascular surfaces, the choroid plexus, perivascular regions, and glial metabolic networks.

This spatial reasoning is essential. If inflammation-related depression is caused by peripheral immune signals that enter or alter the brain via the BBB, brain vasculature, meningeal, or choroid plexus pathways, HCAR1 signaling at these points may influence the conversion of peripheral inflammation into neural dysfunction. The lactate-HCAR1 gate is probably not exclusively found in neurons. It is likely a distributed signaling system that encompasses vascular endothelium, perivascular cells, astrocytes, choroid plexus epithelium, microglia, and neurons, with the vascular-glial interface being a crucial convergence zone.

### 3.3 Distinguishing HCAR1 signaling from other lactate pathways

Mechanistic inflation poses a significant risk in this domain: any effect associated with lactate is easily attributed to “lactate signaling”. To develop a high-resolution model of exercise psychiatry, it is critical to distinguish HCAR1 signaling from at least three other lactate pathways: monocarboxylate transporter-mediated lactate transport, lactate oxidation and mitochondrial adaptation, and lactate-derived protein or histone lactylation. These pathways may interact but are not interchangeable.

#### 3.3.1 Lactate transport via MCT

Monocarboxylate transporters aid in the transport of lactate across cellular and tissue membranes. MCT1, MCT2, and MCT4 have cell-type-specific expression patterns in the central nervous system, allowing lactate exchange between endothelial cells, astrocytes, oligodendrocytes, and neurons (50, 51). According to the astrocyte-neuron lactate shuttle hypothesis, astrocytes produce lactate through activity-dependent glycolysis, which can then be transported to neurons as an oxidative substrate (52). Later research revealed that lactate transport from astrocytes is required for the formation of long-term memory and the preservation of long-term potentiation in the hippocampus (53).

This transport biology is important, but it should not be confused with HCAR1 signaling. MCT-mediated lactate flux characterizes lactate as a substrate and transport molecule, whereas HCAR1 recognizes lactate as a ligand for extracellular receptors. In practice, both pathways may function simultaneously during the exercise. A lactate pulse can increase substrate availability for oxidative cells, alter intercellular lactate gradients, and activate HCAR1-expressing vascular or immune cells. The analytical task is to determine which behavioral or immunological effects require lactate transport, which require receptor activation, or both.

#### 3.3.2 Oxygenation of lactate and mitochondrial adaptation

Lactate acts as a significant oxidative substrate. The lactate shuttle theory states that lactate produced in glycolytic cells can be transported to oxidative cells for energy production (8, 21). Lactate availability in skeletal muscle and the brain is linked to mitochondrial metabolism, redox state, and exercise-induced adaptation (9, 54). Lactate exposure can enhance exercise-induced mitochondrial adaptations by increasing markers like PGC-1α, mitochondrial enzymes, and BDNF-related pathways in specific tissues (54–56).

Mitochondrial adaptation is relevant to inflammation-related depression because fatigue, decreased energy availability, and psychomotor retardation may indicate disrupted bioenergetic control. However, lactate oxidation is conceptually distinct from lactate-HCAR1 signaling. Oxidation investigates the use of lactate as an energy source and its impact on redox and mitochondrial adaptation. HCAR1 signaling investigates whether a receptor detects extracellular lactate and modulates cAMP, inflammatory pathways, vascular responses, and cellular trafficking. A thorough review should avoid conflating “lactate as fuel” and “lactate as receptor ligand” into a single mechanism.

#### 3.3.3 Lactylation: concurrent or subsequent mechanism

Lactylation has added a new dimension to lactate biology. The discovery that lactate-derived histone lysine lactylation can regulate gene transcription in macrophages has elevated lactate from a metabolic intermediate to a potential epigenetic substrate (57). Histone lactylation is thought to influence inflammatory resolution and phenotype modulation in macrophages after glycolysis activation. Recent research suggests that exercise-induced lactate may enhance histone H3 lactylation in microglia, thereby improving cognitive dysfunction and neuroinflammation in murine models. Lactylation has recently been identified as a possible link between metabolism, inflammation, and neurological disorders (58).

Lactylation should be viewed as a highly innovative but still evolving mechanism in exercise psychiatry. The most compelling direct evidence remains preclinical, and lactylation may have context-dependent effects that are not always anti-inflammatory or therapeutic (57–60). Furthermore, lactylation is not the same as HCAR1 signaling. This is an intracellular post-translational modification that affects lactate availability, lactyl-CoA synthesis, and the regulation of chromatin and proteins. In contrast, HCAR1 is a sensory pathway mediated by extracellular GPCRs. Lactylation may occur downstream of exercise-induced lactate, alongside HCAR1, or in completely different cellular environments.

The most robust model is not that exercise lactate acts exclusively through lactylation or exclusively through HCAR1. Rather, exercise generates a lactate pulse that can be interpreted through several mechanistically distinct pathways. MCTs transport lactate across compartments; oxidative tissues use lactate as an energetic substrate; HCAR1-expressing immune and vascular cells detect extracellular lactate as a GPCR ligand; and, in selected cellular contexts, lactate availability may support lactylation-dependent transcriptional or post-translational regulation. These mechanisms may interact, but they should not be treated as interchangeable evidence for a single lactate-HCAR1 pathway. **Table 2** therefore distinguishes HCAR1-dependent, HCAR1-independent, and currently unresolved lactate-related mechanisms relevant to exercise effects in inflammation-related depression.

**Table 2:** Evidence map of lactate-related mechanisms relevant to exercise effects in inflammation-related depression.

Mechanism/pathway	HCAR1 dependence	Primary biological level	Main compartments/cell types	Representative evidence/functional role	Relevance to inflammation-related depression	Human translational support	Main limitations/open questions
Lactate-HCAR1/GPR81 signaling	HCAR1-dependent when receptor activation, blockade, knockdown, or genetic deletion is directly tested	Extracellular receptor-mediated immunometabolic signaling	Bone marrow niche; hematopoietic stem/progenitor cells; monocytes/macrophages; endothelial cells; brain-border interfaces; glia-associated vascular compartments	Links extracellular lactate to GPCR-mediated signaling; implicated in anti-inflammatory responses, vascular adaptation, and exercise-related immune reconfiguration (10–12, 17, 22, 24, 26, 37)	High. This is the central candidate pathway linking exercise-induced lactate, myeloid output, peripheral inflammation, neurovascular interfaces, and neuroinflammatory tone	Low-to-moderate. Supported by receptor biology and preclinical evidence, but direct causal evidence in patients with depression remains limited	Requires cell-specific causal studies, validated HCAR1 agonist/antagonist tools, human immune-cell responsiveness studies, and dose-response validation
MCT-mediated lactate transport	HCAR1-independent	Intercellular substrate shuttling and compartmental lactate transport	Astrocytes; neurons; endothelial cells; skeletal muscle; oxidative tissues	Supports lactate shuttle biology, astrocyte-neuron metabolic coupling, substrate exchange, and memory-related lactate effects (50–53)	Moderate. Important for brain energetics and glial-neuronal metabolic coupling, but less specific for immune-to-brain signaling than HCAR1-mediated pathways	Moderate. Strong physiological basis, but limited depression-specific translation	Difficult to distinguish transport effects from receptor-mediated signaling, lactate oxidation, and downstream metabolic adaptation *in vivo*
Lactate oxidation and mitochondrial adaptation	HCAR1-independent, although downstream adaptation may interact with receptor-mediated signaling	Bioenergetic substrate use and metabolic adaptation	Skeletal muscle; mitochondria-rich tissues; brain; cardiometabolic systems	Lactate serves as an oxidative fuel and may contribute to mitochondrial remodeling, redox regulation, PGC-1α-related adaptation, and energetic resilience (8, 9, 21, 54, 56)	Moderate. May relate to fatigue, low energy, psychomotor slowing, and reduced exercise tolerance in inflammation-related depression	Moderate. Exercise physiology evidence is strong, but psychiatric causality remains indirect	Need clearer links between lactate-driven energetic adaptation and symptom improvement in inflammatory or immune-metabolic depressive phenotypes
Lactylation, including histone/protein lactylation	HCAR1-independent or currently unresolved	Epigenetic or post-translational regulation	Macrophages; microglia; other metabolically responsive immune and neural cells	May connect lactate availability to transcriptional regulation, inflammatory-state remodeling, microglial responses, and neuroinflammatory outcomes (57–60)	Moderate. Mechanistically intriguing, especially for immune and glial reprogramming, but currently more exploratory than HCAR1 signaling	Low. Evidence remains largely preclinical	Timing, cell specificity, causal relevance, directionality of effect, and interaction with HCAR1 signaling remain unresolved
Exercise-induced myokines/adipokines	Non-HCAR1 co-signals; may interact with lactate-dependent pathways	Parallel endocrine-like exercise signaling	Skeletal muscle; adipose tissue; immune cells; vascular and brain-related target tissues	Includes contraction-induced mediators such as IL-6 and adiponectin-related signaling that can shape immune tone, metabolic adaptation, and neuroinflammatory outcomes (31, 61–64)	Moderate-to-high. Likely important co-signals that may cooperate with or modify lactate-HCAR1 effects	Moderate. Repeatedly implicated in exercise biology, but not specific to the lactate-HCAR1 mechanism	Integrated models are needed to determine how myokines/adipokines interact with lactate-HCAR1 signaling across different exercise doses and patient phenotypes
Catecholamines, cortisol, autonomic and thermoregulatory signals	Non-HCAR1 co-signals; may modify lactate kinetics and immune responses	Systemic neuroendocrine and physiological co-signaling	Sympathetic nervous system; HPA axis; circulation; vascular and immune compartments	Contribute to immune redistribution, stress recovery, leukocyte trafficking, inflammatory reactivity, temperature-related responses, and the broader exercise-induced signaling context (32, 33, 45)	Moderate. Likely modifies immune environment, exercise tolerability, stress physiology, and symptom expression	Moderate-to-high. Well established in exercise physiology, but nonspecific for depression mechanisms	Difficult to isolate from lactate effects; these signals may support, amplify, or counter-regulate lactate-HCAR1-related responses
pH-related and interoceptive effects of lactate exposure	HCAR1-independent or unclear	Acid-base physiology, chemosensory signaling, and interoceptive processing	Circulation; respiratory system; brainstem and limbic interoceptive circuits; anxiety-related networks	Lactate exposure can have context-dependent effects; systemic lactate infusion may induce panic-like or aversive interoceptive responses in vulnerable individuals (41)	Moderate. Important boundary condition for patients with anxiety sensitivity, panic vulnerability, hyperventilation tendency, low fitness, or poor recovery capacity	Low-to-moderate. Human panic literature is relevant, but not equivalent to exercise-induced lactate pulses	Need to distinguish adaptive exercise-induced lactate pulses from sustained lactate elevation, impaired clearance, acid-base disturbance, and non-exercise lactate exposure

This table distinguishes receptor-mediated HCAR1/GPR81 signaling from broader lactate biology. MCT-mediated transport, lactate oxidation, lactylation, pH-related effects, and exercise co-signals may interact with HCAR1-dependent mechanisms, but they should not be treated as interchangeable evidence for the lactate-HCAR1 pathway.

## 4 Bone marrow and brain immunity in inflammation-related depression

inflammation-related depression is not adequately represented by a model in which peripheral cytokines simply “enter the brain” and cause depressive symptoms. A more precise framework is required: chronic stress and depression may alter immune-cell production at its source, alter the trafficking and inflammatory properties of circulating myeloid cells, disrupt brain vascular and barrier interfaces, and converge on glial and endothelial nodes that regulate motivational, cognitive, and somatic symptoms (3–5, 65). According to this framework, bone marrow is not a passive hematopoietic reservoir. It is an immunological amplifier that converts stress hormones, sympathetic tone, inflammatory signals, and metabolic cues into altered myeloid output (66–69).

The bone marrow-brain axis is especially important in the study of exercise-lactate biology. To understand exercise-induced lactate-HCAR1 signaling as an immunometabolic gate, it is critical to investigate not only how lactate affects neurons or glia, but also whether it can alter the peripheral immune supply chain, which contributes to neuroinflammation. The bone marrow, circulating monocytes and neutrophils, brain vascular interfaces, choroid plexus, meninges, microglia, astrocytes, and endothelial cells form a decentralized communication network that allows peripheral stress biology to be translated into psychiatric symptoms (16, 69–73, 75, 76).

### 4.1 Stress, depression, and emergency myelopoiesis

Chronic stress can affect hematopoiesis. A seminal study found that chronic variable stress increased the proliferation of hematopoietic stem and progenitor cells, myeloid cell production, and circulating inflammatory leukocytes via sympathetic nervous system signaling within the bone marrow niche (5, 6, 13–15). This discovery altered our understanding of stress-induced inflammation: leukocytosis and monocytosis are not simply side effects of inflammation, but can result from stress-induced changes within the hematopoietic compartment itself.

Social stress models provide additional support for this principle. Social disruption stress increases inflammatory gene expression in leukocytes through β-adrenergic stimulation of myelopoiesis, linking psychosocial adversity to the development of pro-inflammatory myeloid cells (67). Repeated social defeat increases the circulation of bone marrow-derived inflammatory monocytes, which promotes glucocorticoid resistance and the expression of neurovascular adhesion molecules (68). Furthermore, social stress can activate hematopoietic stem and progenitor cells in the bone marrow and spleen, resulting in long-term extramedullary myelopoiesis and an increase in inflammatory susceptibility beyond the acute stress period (69).

These findings are critical for inflammation-related depression because they shift a portion of depression biology beyond the brain. Chronic stress shifts hematopoiesis toward monocytes and neutrophils, predisposing the immune system to inflammatory communication during depressive episodes. The bone marrow acts as a stress-responsive amplifier, converting neuroendocrine and autonomic signals into altered cellular outputs that affect the vascular, meningeal, choroid plexus, and glial systems (66–70).

Data on human depression support this broad immune framework, though direct evidence from patient bone marrow is still limited. Meta-analyses show increased inflammatory markers in major depression, including IL-6, TNF-α, CRP, and other cytokine-related signals. However, there is significant heterogeneity, indicating that inflammation is a biologically relevant subgroup rather than all individuals with depression (3, 4). Recent research on inflammation-related depression has shown that elevated inflammatory biomarkers are common in patients with neurovegetative and motivational symptoms such as anhedonia, fatigue, and psychomotor retardation (5, 6, 13–15). These clinical characteristics are precisely those expected if peripheral immune responses and brain motivational circuits are linked.

The primary implication is that inflammation-related depression should not be viewed solely in terms of cytokine effects on the brain. It may also include stress-induced myeloid bias, which is a change in immune cell production, release, and trafficking that constantly sends inflammatory signals to brain interfaces. This is the point at which bone marrow becomes relevant in exercise psychiatry. If exercise can alter myelopoiesis, monocyte phenotype, or neutrophil trafficking via metabolic signals such as lactate, the antidepressant effect of exercise may be dependent on reprogramming immune supply at the hematopoietic level.

### 4.2 Peripheral myeloid cells are intermediaries between the body and the brain

Peripheral myeloid cells do more than just detect inflammation; they also act as mobile messengers, transmitting stress history from the body to the brain. In social defeat models, Ly6C hi inflammatory monocytes increase and recruit to brain regions involved in threat appraisal and stress processing (70–72). Reducing or obstructing the recruitment of these cells can reduce stress-induced behavioral changes, implying that peripheral monocytes play an active role in neuroimmune signaling (70, 71).

Myeloid cells can affect the brain through a variety of non-exclusive pathways. Initially, they secrete cytokines and inflammatory mediators, such as IL-1β, IL-6, and TNF-related pathways, which can affect endothelial cells, perivascular macrophages, glial cells, and neuronal circuits (3, 65, 71–74). Second, they interact with vascular adhesion molecules and chemokines, which facilitate recruitment to the brain vasculature and perivascular spaces in response to stress (68, 70, 71). Third, they can alter the permeability of the BBB, either directly through endothelial activation or indirectly through cytokine-induced disruption of tight junctions (16, 73, 75, 76). Fourth, they may communicate through brain border structures such as the meninges and choroid plexus, where immune surveillance, cytokine detection, and leukocyte movement are more permissive than at the parenchymal BBB (77–79). Finally, they can initiate or sustain microglial priming, converting transient peripheral inflammation into long-term central immune sensitivity (70–72).

The BBB is an essential component. Chronic social stress weakens the BBB by reducing the tight junction protein claudin-5, particularly in the nucleus accumbens, allowing more peripheral IL-6 and other inflammatory signals to reach reward-related circuitry (16). Previous research has shown that molecular modifications to the BBB can distinguish between stress vulnerability and resilience, implying that the neurovascular unit plays an active role in the transformation of peripheral inflammation into depressive behavior (75). Recent research suggests that claudin-5 and inflammatory cytokines, such as TNF-α, play a role in stress-induced depression (71).

Brain border tissues take this model beyond the traditional BBB. The choroid plexus functions as a blood-cerebrospinal fluid interface, regulating immune cell trafficking, cytokine signaling, and cerebrospinal fluid composition. IFN-γ activates the choroid plexus, which regulates leukocyte infiltration for immune surveillance and repair in the CNS (77). In mood disorders, increased choroid plexus volume has been linked to circulating inflammatory cytokines, suggesting that this structure could serve as an imaging-accessible indicator of altered peripheral-central immune communication (79). The meninges also function as a neuroimmune interface, with resident and recruited immune cells influencing neural function, behavior, and disease susceptibility (78).

Overall, these findings support a model in which peripheral myeloid cells interact with the brain through cytokine secretion, vascular adhesion, barrier modulation, brain-border signaling, and glial priming. This communication does not require extensive leukocyte infiltration of the brain parenchyma. Minor changes in the vascular wall, choroid plexus, meninges, and perivascular niche may be sufficient to alter microglial activity, neurotransmitter metabolism, and circuit functionality.

### 4.3 Microglia, astrocytes, and endothelial cells are convergence points

Peripheral immune signals are not directly related to depressive symptoms. Cells that reside in the CNS and are associated with the brain facilitate their transformation. Three cell types are especially important: microglia, astrocytes, and endothelial cells. Microglia convert inflammatory and stress signals into cytokine synthesis, synaptic restructuring, and circuit-level plasticity; astrocytes manage glutamate clearance, lactate transport, metabolic buffering, and inflammation amplification; and endothelial cells control the interaction of peripheral cytokines and leukocytes with brain tissue through barrier function, adhesion molecules, and vascular signaling (16, 49, 72–76, 79–86).

Tripartite convergence is critical for the lactate-HCAR1 model. Exercise-induced lactate may have an effect on mood without interacting directly with neurons. It may act upstream by affecting myeloid-cell production, at the barrier by influencing endothelial and choroid plexus responses, or within the parenchyma by altering glial inflammatory and metabolic conditions. The relevant target is not a specific cell type, but rather a network of immune-sensitive interfaces.

#### 4.3.1 Microglia: inflammatory priming and synaptic remodeling

Microglia are the CNS’s resident immune cells, but their role in depression goes beyond cytokine production. They investigate synapses, restructure dendritic architecture, modulate complement-dependent pruning, and influence neural circuits throughout childhood and adulthood (79, 81). Microglia actively phagocytose synaptic components and refine postnatal circuits through activity- and complement-dependent processes, according to previous research (79, 81). In pathological cases, the same mechanisms can cause maladaptive synaptic loss or circuit instability.

Models of stress-induced depression demonstrate a link between basic microglial biology and affective symptoms. Social defeat stress causes depressive-like behavior, which is accompanied by microglial activation, peripheral immune recruitment, and neuroinflammatory changes (82). Stress-induced neuronal CSF1 can cause microglia-mediated neuronal remodeling in the medial prefrontal cortex, resulting in synaptic deficits and depressive-like behavior (83). Studies on microglial elimination and repopulation show that stress-induced behavioral consequences are influenced, at least partially, by microglial inflammatory and oxidative conditions (84).

Microglia are at the intersection of immune memory and circuit plasticity. Peripheral myeloid cells and cytokines can sensitize microglia, lowering their threshold for future inflammatory responses (70–72). When activated, microglia can alter synaptic density, dendritic architecture, glutamate equilibrium, and local cytokine levels, thereby altering the functionality of the prefrontal cortex, hippocampus, and reward circuitry (81–84). This implicates microglia as a possible cellular mediator of cognitive decline, increased threat sensitivity, anhedonia, and recurring stress in inflammation-related depression.

#### 4.3.2 Astrocytes’ metabolic buffering and inflammatory amplification

Astrocytes provide an additional point of convergence by integrating metabolism, neurotransmission, and inflammation. They regulate extracellular glutamate, maintain ion and water balance, protect the BBB, supply metabolic substrates to neurons, and participate in inflammatory signaling (50–53, 74, 85, 86). Numerous post-mortem and translational studies have linked major depression to decreased astrocyte quantity, density, or astrocyte-related markers, supporting the idea that astrocyte pathology plays a role in the biology of mood disorders (85).

Astrocytes are especially important in a lactate-centered framework because of the astrocyte-neuron lactate shuttle. Astrocytic glutamate uptake, influenced by activity, can boost glycolysis and lactate secretion, providing neurons with an oxidative substrate during increased synaptic activity (50). This does not imply that all lactate biology in depression is astrocytic; rather, it identifies astrocytes as an important metabolic interface between neural activity and lactate flux.

Inflammation can disrupt this homeostatic function. Inflammatory activation can reduce astrocytic glutamate uptake, increase extracellular glutamate levels, alter metabolic support, and promote excitotoxic or extrasynaptic signaling (74). The interaction of inflammation, glutamate, and glial cells has been identified as a key mechanism in mood disorders, linking peripheral immune activation to synaptic dysfunction and behavioral abnormalities (74). Recent experimental findings suggest that astrocyte reactivity can influence inflammation-induced depression-like behaviors: in mice, ablation of the calcium channel Orai1 in astrocytes reduced inflammatory transcriptional programs, hippocampal inflammatory markers, and LPS-induced anhedonia-like and helplessness-like behaviors (86).

As a result, astrocytes can modulate inflammation by either attenuating or exacerbating it, depending on their condition. In inflammation-related depression, impaired astrocytes may fail to clear glutamate, neglect adaptive lactate shuttling, increase cytokine signaling, and disrupt neurovascular homeostasis. This establishes them as an important collaborator with microglia and endothelial cells in converting peripheral immune stress into depressive symptoms.

#### 4.3.3 Endothelial cells: vascular inflammation, barrier integrity

Brain endothelial cells are the structural and signaling foundation of the BBB. They control paracellular permeability through tight junctions, transcellular transport, leukocyte adhesion, cytokine signaling, and interactions with pericytes and astrocytic endfeet (16, 75, 76). Endothelial dysfunction in depression is more than just a vascular comorbidity; it may also have a direct impact on how peripheral inflammation affects reward, stress, and cognitive circuits.

Chronic social stress provides a compelling experimental example. Depletion of claudin-5 in the nucleus accumbens increases BBB permeability, allowing peripheral inflammatory signals like IL-6 to infiltrate a region important for reward processing and social behavior (16). Human post-mortem and transcriptomic data from studies on stress-related depression highlight the importance of BBB and endothelial modifications in relation to susceptibility and resilience to mood disorders (16, 75). Recent research identifies an EZH2-claudin-5 axis in stress-induced BBB dysfunction, TNF-α infiltration, and depressive-like behavior, placing endothelial tight-junction regulation within the hypothetical integrative pathway linking stress to mood disorders (76).

Endothelial cells act as immune signaling entities. In inflammatory conditions, they express adhesion molecules, chemokines, and cytokine receptors that control leukocyte adhesion and transmigration (68, 70, 71). This allows peripheral myeloid cells to influence the CNS without causing significant parenchymal infiltration. A myeloid cell that interacts with the vascular wall can alter endothelial permeability, activate perivascular macrophages, and elicit responses from astrocytes and microglia. The endothelial layer is more than just a gate that opens and closes; it also serves as a computational surface for interpreting inflammatory, metabolic, and hemodynamic signals.

This point is critical for the lactate-HCAR1 model. HCAR1-mediated effects at vascular or perivascular interfaces may regulate inflammatory trafficking before it progresses to parenchymal neuroinflammation. The vascular-glial unit should be viewed as a primary site of exercise immunometabolic activity, rather than just a passive conduit for peripheral signals entering the brain.

### 4.4 Linking immune changes to dimensions of depressive symptoms

The most robust iteration of an inflammation-related depression model does not claim that inflammation causes “depression” in a general and nonspecific way. It asserts that immune changes have a selective influence on specific symptom dimensions. This distinction is critical for precision exercise psychiatry. If lactate-HCAR1 signaling affects immune response, the clinical effects may be most noticeable not in sadness, but in fatigue, anhedonia, decreased motivation, psychomotor retardation, cognitive inefficiency, and sleep-circadian disruption.

Fatigue can be considered an immune-metabolic symptom. Inflammation redirects energy toward host defense, alters mitochondrial and metabolic signaling, and induces sickness behavior, resulting in decreased activity, low energy, and avoidance of effort. Fatigue in depression may thus indicate a misalignment between peripheral inflammatory burden and central energy allocation mechanisms. Myeloid activation derived from bone marrow may maintain systemic cytokine levels and peripheral immune load, which contribute to this condition (66–70).

Anhedonia is an important symptom dimension in inflammation-related depression. Experimental endotoxin reduces ventral striatal reward responses, providing causal evidence that systemic inflammation can impair neural reward sensitivity in humans (28). In people with depression, inflammatory markers have been linked to reduced corticostriatal reward connectivity and symptoms of anhedonia (29). Inflammatory cytokines can inhibit dopamine synthesis, release, and availability in the basal ganglia and corticostriatal circuits, linking immune activation to impaired reward learning and motivation (6, 29).

diminished motivation and psychomotor retardation are associated with anhedonia, but they prioritize effort, action initiation, and motor performance. Inflammation primarily impairs basal ganglia function and dopaminergic signaling, resulting in decreased motivation to exert effort, impaired movement, and decreased goal-directed behavior (6). These symptoms are particularly relevant to exercise interventions because they predict difficulties in starting exercise and may be among the most biologically responsive to strategies that reduce inflammatory burden or restore dopaminergic-metabolic coupling.

Cognitive slowing can occur through a variety of immune-sensitive mechanisms. Microglial priming can influence synaptic remodeling and plasticity; astrocyte dysfunction can impair glutamate clearance and metabolic support; BBB dysfunction can expose cognitive circuits to inflammatory mediators; and endothelial abnormalities can reduce neurovascular coupling (16, 50–53, 73–76, 79–86). Cognitive symptoms are more than just “top-down” effects of mood disturbances; they may also indicate a breakdown in glial-vascular support for information processing.

Sleep and circadian disruption are both components of the immune phenotype. Sleep, circadian rhythms, and inflammatory signaling are linked: cytokines such as IL-1β and TNF-α impact sleep structure, while sleep disruption and circadian misalignment can increase inflammatory activity (87, 88). Sleep disturbances in depression can both cause and sustain immune activation, resulting in a vicious cycle in which insufficient sleep heightens inflammatory responses, which then exacerbate mood, fatigue, and cognitive function.

These symptom dimensions reveal the translational basis for the bone marrow-brain axis. Chronic stress alters myelopoiesis, circulating myeloid cells transmit inflammatory signals, brain barriers become more permissive, and glial-vascular nodes convert peripheral immune pressure into circuit dysfunction, resulting in inflammation-related depression as a systemic immune communication disorder. The therapeutic potential of exercise-induced lactate-HCAR1 signaling stems from its ability to address underlying conditions rather than directly improving mood; it resets the immune and vascular environments, which influence reward, energy, cognitive, and sleep mechanisms.

## 5 Exercise-induced lactate-HCAR1 signaling: a mechanism for immune reconfiguration

The main point of this review is not that exercise is universally “anti-inflammatory. “ That formulation is overly simplistic for a mechanism-based psychiatry. Exercise is more accurately defined as a regulated physiological disturbance that temporarily alters metabolism, endocrine function, hemodynamics, temperature, immune cell distribution, and tissue-derived signaling molecules (31–33). Lactate is a component of this disturbance, acting as both a metabolic flux marker and a receptor-mediated signaling ligand. Lactate, when produced as a transient exercise pulse, may influence immune cell behavior, vascular regulation, and neuroinflammatory tone via the HCAR1/GPR81 pathway (8–12, 17, 22, 24–26).

This section investigates whether the lactate-HCAR1 pathway can be identified as a mechanism for immune reconfiguration in inflammation-related depression. The available evidence supports this possibility, but with important qualifications. Exercise biology shows that physical activity has a rapid effect on immune distribution and inflammatory thresholds. According to extracellular lactate and HCAR1 biology, lactate can influence macrophages, monocytes, neutrophils, and vascular interfaces. Current depression models provide direct evidence that running exercise can reduce stress-induced myeloid skewing in bone marrow and neuroinflammation through the lactate–HCAR1 axis (11, 12, 17, 22, 24–26, 61–63). Nonetheless, the evidence does not currently support the monocausal model. Exercise produces a variety of concurrent signals, including myokines, catecholamines, cortisol, adipokines, thermoregulatory signals, and lactylation-dependent epigenetic modifications (32, 33, 61–64, 89, 91, 92). The most plausible interpretation is that lactate-HCAR1 functions as a candidate immunometabolic gate: potentially essential in certain contexts, unlikely to be universally adequate, and best understood as part of a larger exercise-induced signaling network.

### 5.1 Results from exercise physiology

Exercise quickly activates immune cells, alters cytokine balance, and modifies the inflammatory reactivity of circulating leukocytes (31, 32). The response includes more than just immunosuppression and anti-inflammation. The direction is determined by exercise intensity, duration, recovery, training status, energy availability, ambient temperature, and baseline inflammatory state (31–33). Acute exercise rapidly mobilizes leukocytes into circulation via catecholamines and shear stress, whereas prolonged or intense exercise activates cortisol and other neuroendocrine mediators, redistributing immune cells and influencing post-exercise inflammatory responses (32). This temporal sequence is significant because the immune system is not simply “turned down”; it is actively trafficked, influenced by metabolic and hormonal signals, and reset during recovery.

Skeletal muscle is essential to this process. Muscle contraction produces myokines and metabolites, which interact with immune, hepatic, adipose, vascular, and neural tissues (33, 61). Interleukin-6 is an excellent example. Unlike infection-induced IL-6, exercise-induced muscle IL-6 increases quickly and promotes anti-inflammatory mediators like IL-1 receptor antagonist, IL-10, and soluble TNF receptors (31, 61–63). This exemplifies a broader principle: the same cytokine can have different biological effects depending on whether it is produced during pathogen-associated inflammation or contraction-induced metabolic demand.

Exercise also generates non-cytokine signals. Lactate levels rise with exercise intensity and metabolic flux; catecholamines regulate immune cell mobilization and tissue trafficking; cortisol aids in immune redistribution during recovery; and body temperature fluctuations can affect monocyte cytokine production and leukocyte activity (32, 33). According to research on heat stress, exercise-induced thermal load can alter hormonal and leukocyte responses, and exercising in heat stress can reduce cytokine production in LPS-stimulated monocytes, implying that temperature and endocrine changes are essential to the immune-modulatory environment (32, 33, 62, 63).

The complexity of this phenomenon demonstrates that exercise-induced immune modulation cannot be reduced to a single anti-inflammatory pathway. Exercise produces a coordinated signal package that includes lactate, myokines, catecholamines, cortisol, hemodynamic shear, temperature, extracellular vesicles, and adipokines (32, 33, 61–64, 89). These signals could converge on myeloid, endothelial, and glial cells. Lactate-HCAR1 is appealing because of its receptor-level specificity in this context; however, its effects should be considered in conjunction with the other exercise signals.

In the context of inflammation-related depression, one important aspect of exercise biology is that repeated sessions can alter the baseline inflammatory state. Consistent physical activity is associated with reduced chronic low-grade inflammation, decreased inflammatory responsiveness, and improved metabolic health (31, 33). However, a candidate mechanism of action for antidepressants may not be limited to a decrease in circulating CRP or cytokines. An advanced mechanism would involve altered immune programming, such as reduced pro-inflammatory myeloid cells, decreased inflammatory inducibility, improved vascular barrier function, and decreased glial priming. Lactate-HCAR1 signaling is one potential mechanism for converting an acute metabolic surge into a long-term immune reconfiguration.

### 5.2 Evidence from lactate and HCAR1 biology

HCAR1/GPR81 converts lactate, a metabolic correlate, into a receptor-mediated signal. HCAR1 was originally identified as a lactate-sensitive GPCR that inhibits adipocyte lipolysis through Gi-coupled cAMP reduction, but it is now linked to immune regulation, vascular adaptation, and neural signaling. This is critical for exercise psychiatry because receptor-defined signaling allows for the formulation of causal questions such as whether lactate modifies inflammatory tone via HCAR1-expressing immune or vascular cells, and whether inhibiting or ablation of this pathway reduces the effect of exercise.

The immune evidence is strongest in innate inflammatory systems. lactate–HCAR1 signaling can reduce inflammation caused by Toll-like receptors and inflammasomes, including pathways involving NF-κB, NLRP3, and IL-1β (11). Lactate suppresses LPS-induced pro-inflammatory cytokine production in macrophages through GPR81-mediated suppression of YAP and NF-κB signaling (24). The findings suggest that extracellular lactate can inhibit excessive innate inflammatory responses, particularly in macrophage-like cells.

Nonetheless, the literature warns against excessive attribution to HCAR1. Lactate can inhibit macrophage inflammatory activation without affecting GPR81 by altering metabolic reprogramming, including changes in LPS-induced glycolysis (25). Exercise-induced lactate is significant because it can modulate immune cells through a variety of pathways, including HCAR1 activation, monocarboxylate transporter-mediated lactate transport, redox changes, mitochondrial oxidation, and intracellular lactylation. As a result, the appropriate question is not “Does lactate inhibit inflammation?” but rather “Which lactate effects are receptor-mediated, metabolic, and cell-state dependent?”.

The evidence from bone marrow highlights the importance of the HCAR1 axis in relation to inflammation-related depression. In inflammatory bone marrow, lactate produced by neutrophils can activate endothelial GPR81, increasing vascular permeability and facilitating neutrophil mobilization (26). This study does not provide a model for exercise or depression; rather, it establishes a fundamental principle: lactate–HCAR1 signaling can modulate myeloid cell release in the bone marrow niche. This is an important mechanistic link in a review on bone marrow-brain immunity. Lactate not only signals immune cell maturation in the bloodstream, but it may also regulate the vascular and stromal pathways that allow myeloid cells to exit hematopoietic niches.

The literature on the brain and vasculature provides an additional connection. Exercise-induced lactate stimulates cerebral VEGF and angiogenesis via HCAR1 (12). HCAR1 is expressed in vascular and pial regions of the brain, and lactate receptor signaling has been linked to neuronal and vascular responses to metabolic activity (12, 37). The findings suggest that lactate-HCAR1 signaling can occur at vascular interfaces that separate blood from brain tissue. In inflammation-related depression, the vascular HCAR1 axis is especially important because it may mediate the effects of peripheral inflammation via endothelial dysfunction, BBB changes, and brain-border immune signaling.

The interplay of lactate and HCAR1 biology suggests four possible mechanisms. Initially, HCAR1 activation may reduce excessive inflammatory responses from macrophages and monocytes (11, 24). Second, lactate may influence myeloid mobilization from bone marrow niches through endothelial GPR81 (26). Third, exercise-induced lactate may influence brain vascular adaptation through HCAR1 (12). Fourth, the effects of lactate may be partially independent of HCAR1, necessitating a fine distinction between receptor-mediated signaling and metabolic and epigenetic lactate pathways (25, 89, 90). This pattern is exactly what one would expect from an immunometabolic gate: specific enough for testing while also being integrated into a larger metabolic network.

### 5.3 Empirical results from depression models

The primary rationale for placing lactate-HCAR1 at the center of this review is the discovery of evidence from depression models linking exercise, bone marrow myelopoiesis, and neuroinflammation. Chronic stress has been shown to stimulate hematopoietic stem and progenitor cells, shifting immune responses toward inflammatory myeloid lineages (66, 67). Social stress models show that bone marrow-derived monocytes can migrate to the brain and influence anxiety and depression-related behaviors (70). These findings identify the primary substrate: stress and depression-like symptoms can alter the immune supply chain before inflammatory signals are transmitted to the brain.

According to studies on depression models, running can reduce neuroinflammation and glial activation. Treadmill running in rats exposed to chronic unpredictable stress restored sucrose preference, decreased the number and activation of hippocampal microglia, and reduced the increase in inflammatory cytokines in the hippocampus (64). Recent research on mice exposed to chronic unpredictable stress found that running exercise reduced depressive-like behavior, altered the microglial M1/M2 balance, and reduced hippocampal neuroinflammation through an adiponectin-AdipoR1-related pathway (89). These studies show that exercise can reduce glial inflammation in depression models, but they do not independently establish lactate-HCAR1 as the causal signal.

A recent study reports a significant mechanistic advancement, indicating that running exercise reduces depression-induced myeloid skewing in bone marrow and neuroinflammation via the lactate–HCAR1 axis (17). In that model, chronic unpredictable stress caused depressive and anxiety-like behaviors, altered hematopoietic stem cell and progenitor dynamics, skewed bone marrow output towards myeloid differentiation, and increased neuroinflammatory signals. Running exercise increased circulating lactate, activated GPR81-related signaling in the relevant hematopoietic compartment, and inhibited stress-induced myeloid-biased differentiation, reducing pro-inflammatory monocyte production and neuroinflammation (17). This represents the most unequivocal evidence available for the model proposed here.

This study is conceptually significant because it moves exercise psychiatry toward a more proactive stage. Rather than simply determining whether exercise reduces microglia activation in the hippocampus, it investigates whether exercise alters the hematopoietic program, which supplies inflammatory cells to both the periphery and the brain. If confirmed, this suggests that exercise-induced lactate can influence processes before the full onset of neuroinflammation, specifically bone marrow output, peripheral myeloid accumulation, and immune-to-brain signaling. This mechanism extends traditional brain-centered accounts by placing hematopoietic programming upstream of neuroinflammation.

#### 5.3.1 Causal evidence

A causal lactate-HCAR1 model requires more than just a correlation between exercise, lactate, and enhanced behavior. Evidence is required at multiple levels: exercise must elevate lactate; exogenous lactate should replicate a portion of the exercise phenotype; HCAR1/GPR81 disruption should reduce the effect; immune reconfiguration should occur upstream or concurrently with neuroinflammatory enhancement; and behavioral recovery should correlate with these immune changes.

The evidence from the depression model is starting to meet this standard. The running-exercise study found a link between exercise-induced lactate and GPR81 activation, which inhibited stress-induced hematopoietic stem cell expansion or myeloid-biased differentiation, decreased pro-inflammatory myeloid output, reduced neuroinflammatory markers, and reduced depressive and anxiety-like behaviors (17). This creates a multi-tiered pathway that links metabolic signaling to hematopoietic outcomes, immune responses, neuroinflammation, and behavior. The design is particularly noteworthy because it goes beyond central cytokines and incorporates bone marrow myelopoiesis into the hypothetical integrative pathway.

Studies unrelated to depression support the validity of receptor-mediated lactate effects. HCAR1 is required for lactate-induced cerebral VEGF and angiogenesis during exercise-related brain adaptation (12). Lactate produced by high-intensity interval training improves mitochondrial function via the GPR81-ERK1/2 pathway, whereas GPR81 knockdown reduces these effects (89). lactate–HCAR1 signaling in innate immunity suppresses inflammatory pathways in macrophages and models of inflammasome-related injury (11, 24). In bone marrow inflammation, endothelial GPR81 promotes lactate-induced neutrophil mobilization (26). These studies do not establish antidepressant efficacy, but they do support the causal relevance of lactate–HCAR1 signaling in vascular, neural, immune, and bone marrow systems.

The most important missing evidence is human translational causality. It remains unclear whether lactate responses during exercise predict symptom amelioration in inflammation-related depression; whether HCAR1 expression or genetic variation influences the antidepressant efficacy of exercise; whether human monocytes or hematopoietic progenitors from depressed individuals exhibit modified HCAR1 responsiveness; or whether lactate-guided exercise can more effectively diminish inflammatory symptom dimensions compared to exercise regimens solely ba These enquiries help to define the discipline’s next phase.

#### 5.3.2 Issue with specificity

A strong model must address the specificity issue, as the effects of exercise cannot be attributed solely to lactate. Exercise alters IL-6 and other myokines, catecholamines, cortisol, adiponectin, extracellular vesicles, heat shock responses, mitochondrial function, vascular shear stress, sleep pressure, and autonomic tone (32, 33, 61–64, 89, 91, 92). Multiple signals have independent effects on immune cells and mood-related circuits. Muscle-derived IL-6 can activate anti-inflammatory mediators, adiponectin can influence microglial polarization in chronic stress models, and exercise-induced lactylation can alter cortical synaptic protein function and improve stress resilience (61–63, 89, 91).

This strengthens rather than weakens the lactate-HCAR1 model. The question concerns whether lactate-HCAR1 is required, adequate, or permissive. An adequate mechanism would be lactate or HCAR1 activation alone, which could replicate the relevant exercise effects on myelopoiesis, neuroinflammation, and depressive-like behavior. A requisite mechanism implies that inhibiting HCAR1 eliminates or significantly reduces exercise-induced immune reconfiguration. A permissive mechanism implies that lactate-HCAR1 promotes immune changes but requires co-signals from myokines, catecholamines, cortisol, or adipokines to achieve the full antidepressant effect.

Current evidence supports a necessary-or-permissive interpretation in specific preclinical models, rather than universal sufficiency. Lactate and GPR81 manipulations can replicate or disrupt selected aspects of exercise adaptation in brain, immune, or metabolic systems (11, 12, 17, 24, 92). However, some lactate effects are independent of GPR81, and some antidepressant-relevant effects of exercise are mediated by non-lactate pathways, including adiponectin-AdipoR1 signaling, myokine responses, mitochondrial adaptation, behavioral reinforcement, and lactylation-associated mechanisms (25, 89, 91). Therefore, lactate-HCAR1 should not be presented as the sole or established mediator of exercise’s antidepressant effects. It is better described as a candidate immunometabolic gate within a broader multi-signal exercise-response network.

A pharmacological caveat exists. Certain studies have used 3-hydroxybutyrate derivatives or analogous compounds as potential GPR81 antagonists; however, concerns have been raised about the validity of 3-OBA as a GPR81 antagonist (93). This is significant because strong causal claims require precise genetic, pharmacological, and cell-specific instruments. Future research should focus on conditional Hcar1 deletion in hematopoietic, endothelial, and brain-border compartments; receptor-rescue methodologies; validated agonists or antagonists; and ex vivo assessments of human immune cell responsiveness to exercise-induced lactate concentrations. This specificity issue also raises the question of whether non-exercise lactate-based interventions could reproduce exercise-like immune or behavioral effects. Potential approaches include lactate administration, lactate salt supplementation, dietary or microbiome-based strategies that alter lactate availability, and pharmacological HCAR1 activation (22, 90). These approaches would be useful experimentally because they could help determine whether lactate is a mediator, a biomarker of exercise exposure, or a permissive signal within a broader exercise response. However, non-exercise lactate exposure should not be assumed to be equivalent to exercise-induced lactate pulses. Exercise simultaneously produces myokines, catecholamines, cortisol changes, vascular shear stress, thermoregulatory responses, mitochondrial adaptation, autonomic changes, and behavioral reinforcement. Moreover, systemic lactate infusion may produce aversive interoceptive or panic-like responses in vulnerable individuals (41, 94). Therefore, non-exercise lactate interventions may reproduce only selected components of exercise biology and should be evaluated as mechanistic probes rather than direct substitutes for exercise.

### 5.4 Pathway integration: lactate-HCAR1 immunometabolic gate model

**Figure 1:**
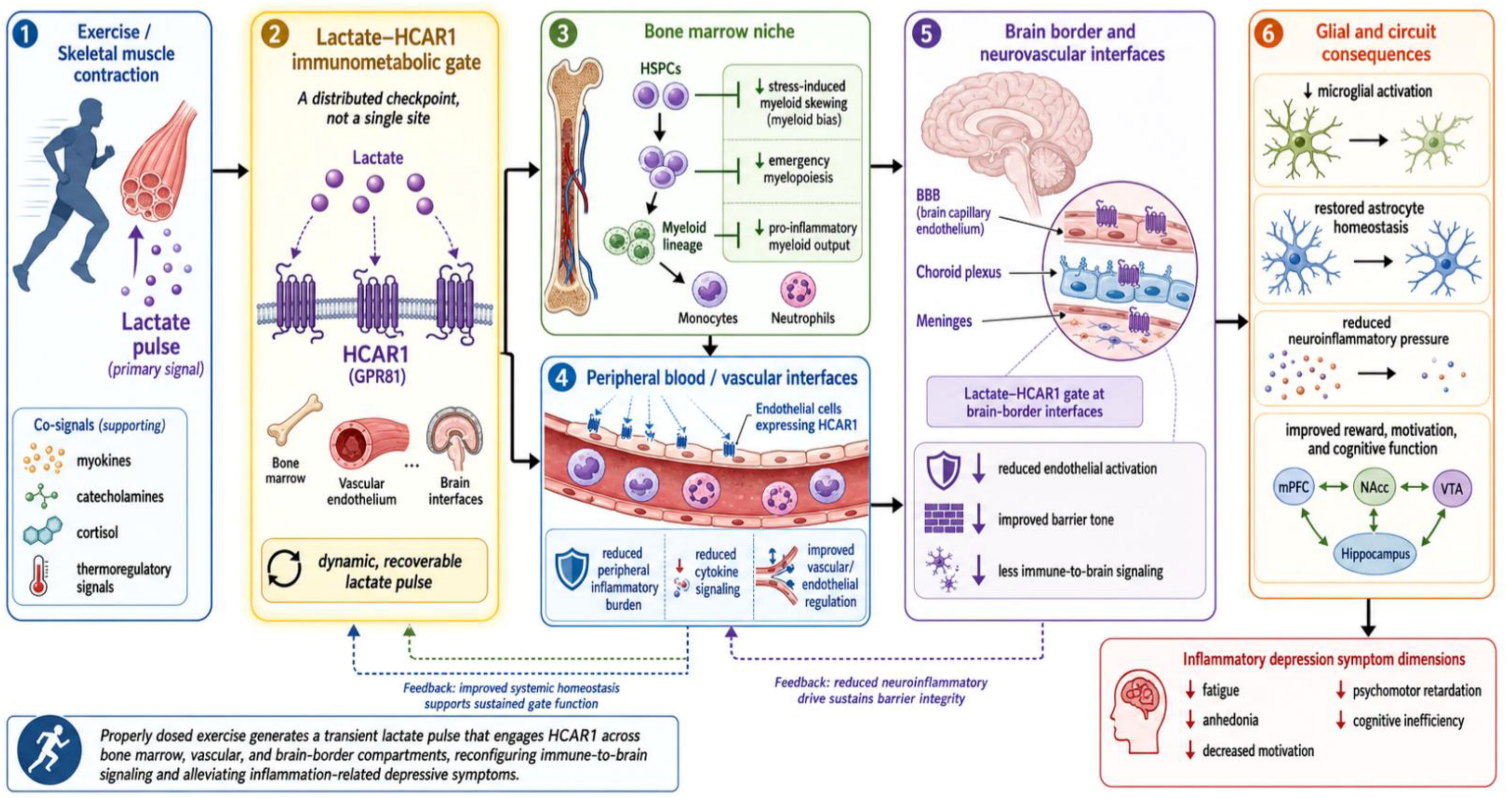
Hypothetical lactate-HCAR1 immunometabolic gate linking exercise to bone marrow-brain immune regulation in inflammation-related depression. Properly dosed exercise may induce a transient and recoverable lactate pulse from contracting skeletal muscle. Beyond serving as an energetic substrate, lactate may act as a receptor-mediated immunometabolic signal through hydroxycarboxylic acid receptor 1 (HCAR1/GPR81). In this proposed model, exercise-induced lactate may engage a distributed HCAR1-dependent gate across bone marrow niches, circulating myeloid cells, vascular endothelium, and brain-border interfaces. Within the bone marrow, lactate-HCAR1 signaling may contribute to the regulation of stress-induced myeloid skewing, emergency myelopoiesis, and pro-inflammatory monocyte/neutrophil output. In the peripheral circulation and vascular compartments, this pathway may influence cytokine signaling, endothelial activation, and inflammatory trafficking. At brain-border and neurovascular interfaces, including the blood-brain barrier, choroid plexus, and meninges, lactate-HCAR1 signaling is proposed to modulate immune-to-brain inflammatory transmission and barrier-related signaling. These peripheral and interface-level effects may reduce neuroinflammatory pressure on microglia, astrocytes, endothelial cells, and reward-, motivation-, and cognition-related circuits. This cross-organ hypothesis may be particularly relevant to inflammation-related depressive symptom dimensions, including fatigue, anhedonia, diminished motivation, psychomotor slowing, and cognitive inefficiency. HCAR1, hydroxycarboxylic acid receptor 1; GPR81, G protein-coupled receptor 81; HSPCs, hematopoietic stem and progenitor cells; BBB, blood-brain barrier; mPFC, medial prefrontal cortex; NAcc, nucleus accumbens; VTA, ventral tegmental area. This figure represents a hypothetical integrative model rather than a fully validated causal pathway. The strength of evidence differs across compartments, with stronger support for exercise-induced lactate kinetics and selected preclinical HCAR1-dependent mechanisms, but weaker direct evidence for the complete pathway in patients with depression.

We present a hypothetical integrative model in which exercise-induced lactate pulses may contribute to immune and neurovascular regulation through a distributed lactate-HCAR1 immunometabolic gate (**Figure 1**).

This model starts in skeletal muscle and does not end in the brain. Lactate levels rise briefly during properly dosed exercise and are detected by HCAR1-expressing cells in immune, vascular, and brain compartments (10–12, 17, 22, 24–26). HCAR1 signaling in the bone marrow may influence the behavior of hematopoietic stem and progenitor cells, the function of the endothelial niche, and the release of myeloid cells (17, 26, 66). Lactate-HCAR1 may reduce excessive inflammatory activation of macrophages and monocytes in the blood and peripheral tissues (11, 24). HCAR1-related signaling at brain vascular and border interfaces may influence endothelial function, angiogenic adaptation, barrier tone, and the transmission of peripheral inflammatory signals to glia (12, 24, 37, 73, 75–79). The subsequent outcome in the central nervous system could include reduced microglial activation, altered cytokine levels, and increased support for reward, energy, and cognitive pathways (64, 89).

The model is deliberately gate-based rather than pathway-linear. A single linear pathway would imply that lactate travels directly from muscle to brain, resulting in antidepressant effects. The evidence suggests that lactate may function at various immune checkpoints, with the psychiatric effect resulting from a reorganization of inter-organ communication. The production of bone marrow, the circulating myeloid phenotype, endothelial barrier function, and microglial state are all interconnected mechanisms that act as sequential filters, translating chronic stress into symptoms and allowing exercise to restore resilience.

This framework explains the significance of lactate dosage and timing. A transient lactate pulse may activate adaptive HCAR1 signaling and aid in immune recalibration, whereas chronic lactate elevation in metabolic diseases or inflammatory conditions may indicate ongoing glycolytic stress and may not elicit the same receptor state or cellular response. The psychiatric goal is therefore not “increased lactate,” but rather a consistent, recoverable, and cell-interpretable lactate signal. This point is critical for future clinical applications: lactate should be viewed as a dynamic metabolic dose rather than a static biomarker.

For the central figure, the pathway is represented as a cross-organ hypothesis rather than as a validated linear mechanism. Skeletal muscle contraction produces lactate together with myokines, catecholamines, cortisol changes, vascular shear stress, and thermoregulatory signals. Lactate may engage HCAR1 in bone marrow niches, circulating myeloid cells, and vascular interfaces, thereby contributing to reduced myeloid-biased output or inflammatory signaling in selected contexts. Brain endothelial cells, choroid plexus and meningeal interfaces, astrocytes, and microglia are proposed downstream sites where altered peripheral inflammatory pressure may influence neuroinflammatory tone. The model links these biological changes to symptom dimensions such as fatigue, anhedonia, diminished motivation, psychomotor slowing, and cognitive inefficiency. This illustration is intended to guide mechanistic testing, not to imply that all steps have been established in humans.

## 6 A research agenda for lactate-informed exercise prescription in inflammation-related depression

**Figure 2:**
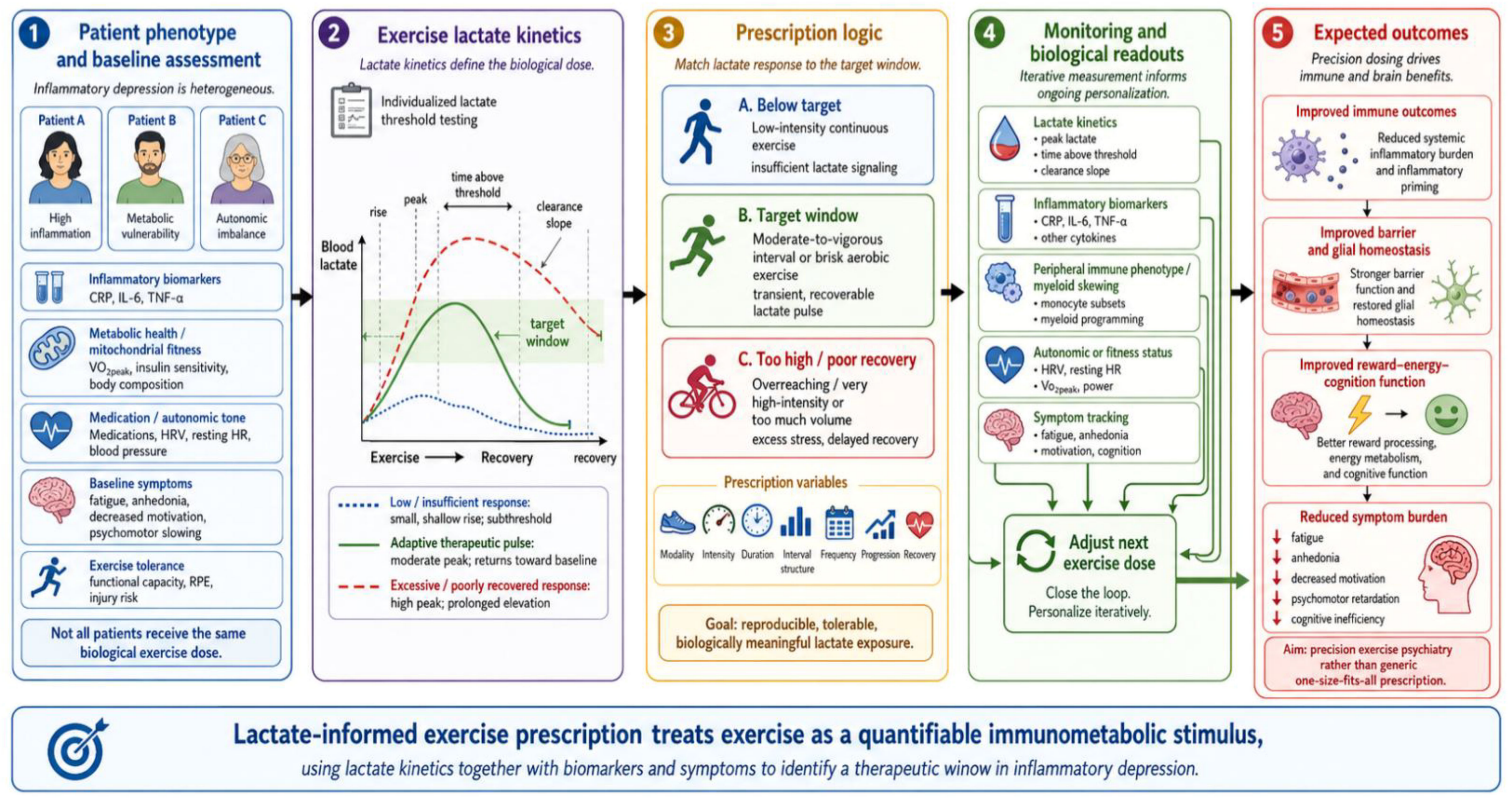
Proposed research framework for lactate-informed exercise prescription in inflammation-related depression. This image illustrates a proposed translational research framework for testing whether lactate kinetics can help personalize exercise prescription in inflammation-related depression. Patients with depression may differ in inflammatory burden, metabolic health, autonomic tone, medication status, baseline symptoms, anxiety sensitivity, fitness level, and exercise tolerance. Therefore, the same nominal exercise program may produce different biological lactate exposures across individuals. Individualized lactate assessment may help distinguish three response patterns: a low or insufficient lactate response, a transient and recoverable lactate pulse within a hypothesized adaptive window, and an excessive or poorly recovered response characterized by a high peak, prolonged exposure, or delayed clearance. In this model, the proposed goal is not maximal lactate accumulation, but a reproducible, tolerable, and recoverable lactate pulse that may engage immunometabolic signaling without imposing excessive physiological or interoceptive stress. Exercise variables, including modality, intensity, duration, interval structure, frequency, progression, and recovery, could be adjusted in future trials to test whether lactate exposure can be maintained within this hypothesized adaptive window. Iterative monitoring of lactate kinetics, inflammatory biomarkers, peripheral immune phenotypes, autonomic or fitness status, safety, adherence, and symptom dimensions may guide dose refinement across sessions. Hypothesized outcomes to be tested include reduced systemic inflammatory burden, improved vascular and brain-barrier regulation, better glial homeostatic support, enhanced reward-, energy-, and cognition-related function, and lower inflammation-related symptom burden, including fatigue, anhedonia, diminished motivation, psychomotor slowing, and cognitive inefficiency. CRP, C-reactive protein; IL-6, interleukin-6; TNF-α, tumor necrosis factor-α; HRV, heart-rate variability; RPE, rating of perceived exertion; VO₂peak, peak oxygen uptake. This framework is proposed for future clinical testing and should not be interpreted as an established clinical prescription algorithm. Its validity depends on prospective studies showing that lactate kinetics predict biological target engagement, symptom improvement, safety, tolerability, adherence, and added value beyond conventional heart-rate-, VO₂-, or RPE-based exercise prescription methods.

The lactate-HCAR1 framework should be viewed as a research agenda for mechanism-based exercise trials rather than as a clinically validated prescription strategy. Most depression studies prescribe exercise according to frequency, duration, and broad intensity categories, such as minutes per week, session count, percentage of maximal heart rate, or perceived exertion. Individualized lactate assessment may help distinguish three response patterns: a low or insufficient lactate response, a transient and recoverable lactate pulse within a hypothesized adaptive window, and an excessive or poorly recovered response characterized by a high peak, prolonged exposure, or delayed clearance (**Figure 2**). These variables are clinically useful, but they may not capture the biological exposure produced by exercise. If exercise-induced lactate contributes to immune reconfiguration, two patients assigned to the same nominal “moderate” or “vigorous” intervention may experience different immunometabolic signals (1, 18, 34). One person may remain below a lactate threshold, another may produce a transient recoverable lactate pulse, and another may accumulate lactate with delayed recovery because of low fitness, metabolic disease, medication effects, poor sleep, or autonomic dysregulation.

A lactate-informed model does not imply that patients with depression should perform high-lactate exercise. It implies that lactate kinetics—rise, peak, duration, area under the curve, and recovery—can be measured as biological features of an exercise session in future trials. This approach may be especially relevant to inflammation-related depression, where the target outcomes may include fatigue, anhedonia, diminished motivation, psychomotor slowing, cognitive inefficiency, and disrupted sleep-wake regulation rather than only total depression severity (4–6, 15). The goal is not to replace conventional exercise prescription, but to test whether lactate-informed dosing adds predictive or mechanistic value beyond heart rate, VO_2_, perceived exertion, and standard clinical monitoring.

### 6.1 Patient stratification: identification of potential beneficiaries

Future trials should not treat major depression as a single exercise-responsive cohort. The lactate-HCAR1 model is most credible in patients whose depression is accompanied by peripheral inflammation, metabolic dysregulation, or somatic-motivational symptoms. This is not to say that other patients cannot benefit from exercise; rather, it suggests that a candidate mechanism, dosage, and expected clinical outcomes may differ across subgroups.

The initial cohort includes patients with elevated inflammatory biomarkers. Meta-analysis shows that certain subsets of depression patients have elevated levels of inflammatory markers like CRP, IL-6, and TNF-α, with significant individual differences (4). This variability explains why biomarker stratification is important. Inflammatory enrichment has shown promise in treatment studies: TNF antagonism did not have broad antidepressant efficacy in treatment-resistant depression; however, patients with elevated baseline inflammatory markers experienced more significant symptom improvement (95). Treatment-resistant depression is associated with elevated peripheral CRP levels and extensive inflammatory activation, suggesting that inflammation may define a subgroup that traditional monoaminergic therapies do not adequately address (96, 97).

Patients in the second cohort experience fatigue, anhedonia, and decreased motivation. These symptoms are more than just indicators of severity; they represent biologically informative dimensions. Inflammation primarily affects corticostriatal reward and motor circuits, partly through effects on dopamine availability, effort valuation, and basal ganglia functionality (6, 28–30). Patients with predominant effort avoidance, psychomotor retardation, and reduced reward sensitivity may benefit from exercise interventions that improve immune-metabolic communication rather than addressing only cognitive mood symptoms.

The third category includes patients with metabolic syndrome, obesity, or insulin resistance. Depression frequently coexists with immunometabolic abnormalities, and people who are depressed and have high levels of inflammation are more likely to develop obesity or metabolic syndrome (15, 98). Lactate-guided exercise is appealing to this group because it can improve inflammatory load, cardiorespiratory fitness, insulin sensitivity, and depressive symptoms all at the same time. Nonetheless, this group may have significant lactate kinetic variations due to decreased oxidative capacity, altered muscle metabolism, and impaired recovery. Lactate measurement may be especially useful for these patients in avoiding both underdosing and overdosing.

Patients in the fourth group do not respond well to conventional antidepressants. Inflammatory markers have been linked to antidepressant resistance (95, 97). If chronic inflammation impairs monoaminergic, glutamatergic, or reward-circuit function, exercise may serve as an additional immunometabolic intervention. The goal is not to replace pharmacotherapy, but to identify a subgroup for whom exercise is recommended as a biological supplement with quantifiable immune and metabolic outcomes.

A pragmatic stratification model may therefore include three tiers. Fatigue, anhedonia, psychomotor retardation, diminished motivation, hypersomnia, and somatic manifestations are among the initial clinical phenotypes. The second assessment focuses on blood biomarker levels (CRP, IL-6, TNF-α) and metabolic indicators (fasting glucose, insulin resistance, lipids, and adiposity). The exercise-metabolic phenotype includes baseline cardiorespiratory fitness, lactate threshold, lactate clearance, and tolerance to exertional interoceptive signals. Patients with convergence across these layers may be the best candidates for lactate-guided exercise trials. Important sources of variability should be measured rather than treated as background noise. Lactate responses to exercise are strongly influenced by fitness level, exercise intensity, mitochondrial capacity, and lactate clearance kinetics (18, 34–36). In clinical depression trials, additional factors such as age, sex, obesity, insulin resistance, medication use, sleep quality, diet, autonomic tone, anxiety sensitivity, and inflammatory burden should also be measured because they may modify exercise tolerance and immune-metabolic responses (31–33). These factors may influence both the magnitude of lactate production and the speed of lactate clearance. They may also determine whether a given exercise dose is experienced as adaptive, neutral, or excessively stressful. Future trials should therefore stratify or adjust for these variables when testing lactate-informed exercise prescriptions.

### 6.2 Developing lactate-informed exercise interventions

An exercise prescription that considers lactate exposure would not begin only with the question, “How many minutes per week?” It would also ask a more mechanistic question: “What metabolic signal does this exercise session produce in this patient?” Blood lactate testing is already used in exercise physiology and clinical exercise assessment to determine intensity, threshold behavior, and recovery. The goal in psychiatry is to test whether this rationale can improve mechanism-based depression trials.

The fundamental design principle is that lactate should be viewed as a dosage signal rather than an optimization target. An effective lactate response requires a moderate-to-vigorous stimulus that exceeds the individual’s low-lactate steady state while remaining below the threshold that causes panic, pain, sleep disturbances, excessive cortisol release, injury, or noncompliance. This differentiation is critical. Instead of persistent lactate elevation or maximal exertion, the proposed therapeutic entity is a reproducible and recoverable lactate pulse, also known as a “lactate window”.

A lactate-informed trial could thus determine exercise dosage using a variety of metrics, including pre-exercise lactate, peak post-exercise lactate, time to peak, area under the lactate curve, recovery slope, heart-rate-lactate coupling, perceived exertion-lactate discrepancy, and day-to-day reproducibility. These variables would allow researchers to distinguish between patients who are under-stimulated and those who are metabolically overstressed by the same nominal program.

#### 6.2.1 Comparing moderate continuous training to high-intensity interval training

Moderate-intensity continuous training is expected to remain the most safe and scalable initial treatment option for a large number of depressed patients. It is easier to implement, causes less musculoskeletal and interoceptive strain, and may be more appealing to sedentary or medically complex patients. It can improve depressive symptoms, cardiorespiratory fitness, and metabolic health, allowing for a gradual transition to personalized threshold-based dosing.

High-Intensity Interval Training (HIIT) is biologically appealing because it causes distinct lactate surges in a short period of time. This makes it a useful tool for studying lactate-HCAR1 mechanisms. Nonetheless, HIIT should not be assumed to be more effective for inflammation-related depression simply because it produces more lactate. Meta-analytic evidence on high-intensity interval training (HIIT) and depressive symptoms is inconsistent and context-dependent, with some analyses indicating minimal or low-certainty effects in healthy populations, while more recent studies suggest modest benefits in people with depression, albeit with small sample sizes and heterogeneity (99, 100). Mixed evidence supports the lactate-window model: high-intensity exercise can be beneficial when it produces a manageable, recoverable signal, but it is harmful when it exceeds psychological or physiological limits.

The psychiatric caveat is significant. High-Intensity Interval Training produces strong interoceptive sensations such as tachycardia, dyspnea, heat, muscle fatigue, and discomfort caused by lactate accumulation. These sensations can be distressing or destabilizing in people who have panic sensitivity, trauma-induced hyperarousal, significant anxiety, or reduced exercise capacity. As a result, HIIT should only be used after assessing anxiety sensitivity, cardiovascular risk, baseline fitness, sleep stability, and patient preferences. A graduated model could improve safety: start with moderate continuous exercise, assess lactate threshold and recovery, and then add brief intervals only when the patient can tolerate exertional signals.

#### 6.2.2 Resistance training and lactate signaling

Resistance training should not be viewed as inferior to aerobic exercise. Independent evidence supports the antidepressant effects of resistance exercise training, including meta-analytic data that show a decrease in depressive symptoms across randomized trials (101, 102). Recent studies of clinically diagnosed depression support resistance training as a potentially effective intervention, though heterogeneity and variation in prescription remain significant factors (102).

Resistance training is relevant to lactate physiology. Resistance training can result in significant lactate accumulation, depending on the load, volume, rest interval, muscle mass engaged, and proximity to fatigue (103, 104). Reduced rest intervals, increased volume, and multi-joint exercises usually increase metabolic stress and lactate responses, whereas low-volume, strength-oriented protocols may produce lower lactate signals. This suggests that resistance training can improve strength and self-efficacy while also producing regulated lactate pulses in patients who are unable to run or cycle.

This point has clinical significance. Many patients with depression have low motivation, obesity, pain, poor cardiorespiratory fitness, or anxiety about aerobic activity. Resistance training may be preferred due to its intermittent nature, structured format, scalability, and psychological reinforcement. It also makes it easier to control lactate exposure using sets, repetitions, rest intervals, and muscle group selection. Resistance training, within a lactate-guided framework, is more than just an alternative exercise modality; it is a distinct metabolic prescription platform.

#### 6.2.3 Temporal considerations, recovery, and adaptation

The therapeutic signal includes more than just the lactate peak. Recovery may have equal significance. A sharp lactate peak followed by rapid clearance may indicate strong oxidative capacity and metabolic adaptability, whereas a moderate peak followed by slow clearance may indicate inadequate conditioning, metabolic strain, or compromised recovery. As a result, blood lactate kinetics can help distinguish between an adaptive and an excessive exercise session (18, 34).

Timing is also important for psychiatric outcomes. Exercise late in the evening may disrupt sleep in some people; exercising during periods of significant fatigue or acute stress may increase perceived strain; and high-intensity workouts without adequate recovery may exacerbate inflammatory load, soreness, or avoidance behaviors. The lactate-window model suggests that training regimens should include a recovery framework that includes high-lactate sessions, low-intensity recovery days, sleep assessment, and progression only after consistent recovery responses.

Repeated exposure is probably required. A single lactate pulse may temporarily alter immune signaling; however, long-term antidepressant effects are likely dependent on repeated exposures that promote adaptation in muscle oxidative capacity, inflammatory responsiveness, endothelial function, autonomic regulation, and glial-vascular tone. The goal is to avoid causing episodic metabolic shock and instead improve the system’s immune-metabolic flexibility.

### 6.3 Biomarkers for mechanism-based trials

Numerous exercise trials in depression have a fundamental limitation in that they focus on symptom assessment rather than evaluating biological dosage or underlying mechanisms. Trials based on lactate should be structured differently. They should link exercise exposure, lactate kinetics, immune reconfiguration, neuroinflammatory indicators, reward circuitry, and symptom dimensions in the same subjects. A minimal clinical validation design would include: baseline inflammatory stratification using CRP, IL-6, TNF-α, and metabolic markers; graded exercise testing to determine fitness and lactate threshold; serial lactate sampling before exercise, immediately after exercise, and during recovery; assessment of leukocyte subsets and myeloid-cell phenotypes; dimensional symptom measures for fatigue, anhedonia, motivation, psychomotor slowing, sleep, and cognition; safety monitoring for panic symptoms, excessive fatigue, sleep disruption, and non-adherence; and comparison with conventional heart-rate-, VO_2_-, or perceived-exertion-based exercise prescription. This design would test whether lactate kinetics predict biological and clinical response rather than assuming that lactate is already a validated dosing target.

Initially, trials should evaluate blood lactate trajectories rather than individual post-exercise measurements. The minimal design would include resting lactate, immediate post-exercise lactate, peak lactate timing, and recovery values at predetermined intervals. More sophisticated designs could calculate lactate area under the curve, clearance half-time, and their relationship to heart rate and perceived exertion (18, 34). These measurements would allow researchers to determine whether the lactate response predicts antidepressant efficacy or immune alteration.

Second, trials should evaluate peripheral inflammatory markers like CRP, IL-6, and TNF-α (4, 5). C-reactive protein (CRP) may be particularly useful for stratification due to its clinical availability, despite being nonspecific and influenced by factors such as obesity, infection, smoking, and medical comorbidities. Cytokines provide more mechanistic clarity, but they require meticulous assay standardization and consideration of circadian rhythms, fasting conditions, and recent physical activity.

Third, trials should characterize myeloid cells. Flow cytometry or single-cell methodologies could be used to quantify classical, intermediate, and non-classical monocytes, neutrophil activation, myeloid-derived inflammatory signatures, and adhesion molecule or chemokine receptor expression. If the bone marrow-brain model is correct, exercise should result in not only lower CRP levels, but also altered myeloid cell states and trafficking capabilities.

Fourth, the trials must evaluate HCAR1-related biology. This could include HCAR1 expression in peripheral blood mononuclear cells or isolated monocyte populations, lactate-induced ex vivo cytokine responses, cAMP-related measurements, GPR81 pathway indicators, and possibly genetic variations in HCAR1 or related signaling components (11, 12, 24, 26). Due to the inadequacy of pharmacological tools for HCAR1, ex vivo functional assays may prove especially beneficial.

Fifth, trials must include cerebral biomarkers. TSPO PET has been used to assess neuroinflammation-associated signals in major depression; however, interpretation is difficult because TSPO is non-specific to microglia and is modulated by genotype, cell type, and ligand characteristics (105, 106). fMRI reward tasks and resting-state corticostriatal connectivity may provide more scalability for linking inflammation to anhedonia and motivation (29, 30). An extensive trial would investigate whether lactate-guided exercise affects both inflammatory markers and reward-circuit functionality.

Ultimately, clinical outcomes should be dimensional. Standard depression scales are necessary but inadequate. Trials must include precise measures of fatigue, anhedonia, effort-based decision-making, psychomotor retardation, sleep quality, and cognitive efficiency. The following symptoms are most directly predicted by the inflammation-related depression model (6, 15, 29, 30). A low total depression score may mask a significant impact on fatigue or anhedonia; however, mood enhancement without changes in immune function would contradict the proposed mechanism.

### 6.4 Risks, limitations, and psychiatric issues

The lactate-HCAR1 model is only clinically useful if its boundaries are clearly defined. Lactate should not be regarded as universally beneficial. Sodium lactate infusion has been used as an experimental panicogenic challenge in panic disorder, with affected individuals being more susceptible to lactate-induced panic than control subjects (107). This does not imply that exercise-induced lactate is the same as intravenous lactate infusion; rather, it suggests that lactate-rich exercise regimens should be approached with caution in people who have panic sensitivity, severe anxiety, interoceptive fear, or tendencies to hyperventilation.

Bipolar disorder is an additional boundary condition. Exercise can improve mood, sleep, and metabolic health in bipolar disorder, but the relationship between physical activity, activation, sleep, and mood polarity is complex (94). High-intensity exercise that disrupts sleep, increases goal-directed activation, or reinforces excessive activity patterns may destabilize susceptible patients. Lactate-guided exercise should be incorporated into sleep-wake stabilization, mood monitoring, and careful progression for patients with bipolar depression, especially when vigorous intervals are included.

Metabolic disorders complicate the interpretation. Individuals with obesity, insulin resistance, metabolic syndrome, or metabolic dysfunction who take antipsychotic medications may have altered lactate production, clearance, and oxidative capacity (15, 98, 108). In these patients, the same external workload may result in increased perceived exertion, lactate accumulation, or longer recovery times. lactate-informed dosing in future trials may be especially useful in this situation; however, it should be introduced gradually and accompanied by metabolic monitoring.

Overtraining poses a contrasting risk. If the lactate-window model is correct, increased intensity is not necessarily advantageous. Excessive training load, insufficient recovery, sleep deprivation, and psychological stress can all cause maladaptive neuroendocrine and immune changes, resulting in fatigue, mood swings, and inflammatory dysregulation (109). A significant increase in exercise may worsen adherence issues and symptoms in patients with depression who already have low energy and high inflammatory levels. As a result, recovery metrics should be built into the prescription rather than added as an afterthought.

Medication effects are another understudied topic. Antidepressants, antipsychotics, mood stabilizers, and beta-blockers can all have an effect on autonomic responses, thermoregulation, weight, glucose metabolism, sedation, motivation, and exercise tolerance. The impact of psychiatric medications on physical performance metrics is variable and not completely understood (108). The significance stems from the fact that lactate response is influenced not only by exercise intensity but also by physiological factors, pharmacological interventions, sleep quality, nutritional status, and metabolic health. Future trials should document and analyze the effects of medication rather than treating them as extraneous variables.

### 6.5 Prospective directions

The lactate-HCAR1 framework outlines a distinct translational research agenda. The discipline should move from asking whether exercise relieves depression to looking into which biological signals from exercise enhance specific depressive phenotypes in individual patients.

#### 6.5.1 Is HCAR1 required for the antidepressant effects of exercise?

The first question concerns necessity. Does the absence of HCAR1 signaling eliminate or reduce the antidepressant, anti-inflammatory, or bone marrow-rewiring effects of exercise? Preclinical evidence currently links running exercise to lactate, GPR81, bone marrow myeloid skewing, and neuroinflammation in depression models (17). Nonetheless, more robust causal investigations are needed, including conditional Hcar1 deletion in hematopoietic cells, endothelial cells, and brain-border compartments; rescue experiments; validated agonists and antagonists; and cell-specific transcriptomic analyses.

This question is also relevant to human studies. Human studies should investigate how baseline HCAR1 expression, lactate-induced immune responses, and HCAR1 genetic variation affect clinical responses to exercise. In the absence of this step, HCAR1 will remain a viable mechanism rather than a confirmed treatment-relevant target.

#### 6.5.2 Can lactate response predict clinical improvement?

The next question concerns prediction. If lactate acts as a biological dosage signal, the lactate response should predict the degree of clinical or immune enhancement. This does not necessitate a simple linear model in which elevated lactate is consistently associated with increased benefit. A more likely pattern is an inverted-U or window effect: insufficient lactate may not activate immunometabolic signaling, whereas excessive or inadequately recovered lactate may cause stress, discomfort, or noncompliance.

Subsequent trials should compare standard exercise prescription to lactate-guided prescription. The primary goal will be to determine whether lactate-guided dosing improves inflammatory symptoms, reward-circuit functionality, or myeloid-cell phenotype more than heart-rate-based dosing.

#### 6.5.3 Is it possible to apply peripheral immune rewiring to human psychiatry?

The third inquiry focuses on the translation of animal models to human patients. The bone marrow-brain axis is well established in stress biology, but direct evidence in humans for depression is still limited. Human studies face difficulties in repeatedly sampling bone marrow; however, they can estimate peripheral immune output using monocyte and neutrophil phenotyping, inflammatory transcriptomics, cytokine panels, soluble adhesion molecules, and imaging of neuroinflammatory or reward-circuit alterations.

A robust translational trial would evaluate the entire pathway: exercise-induced lactate pulse → peripheral HCAR1-mediated immune response → myeloid phenotype change → decreased inflammatory biomarkers → altered reward or neuroinflammation imaging → increased fatigue, anhedonia, and low motivation. This design would shift the field from association to mechanism.

#### 6.5.4 Can exercise be studied as a quantifiable metabolic intervention?

Exercise is frequently described as a form of medicine, but the analogy should not be overstated. Unlike a drug, exercise is a multi-component behavioral and physiological intervention that produces metabolic, endocrine, autonomic, vascular, thermoregulatory, immune, and psychological effects simultaneously. A lactate-informed framework may nevertheless allow exercise to be studied with some pharmacodynamic concepts, including biological exposure, peak response, recovery, target engagement, response heterogeneity, and adverse effects.

Future trials should therefore determine whether lactate-informed prescription improves mechanistic or clinical outcomes beyond conventional approaches. A validated prescription model would need to show that lactate kinetics predict changes in inflammatory biomarkers, myeloid-cell phenotypes, reward or neuroinflammatory readouts, and symptom dimensions. It would also need to show safety, tolerability, adherence, and superiority or added value compared with heart-rate-, VO_2_-, or perceived-exertion-based prescriptions. Until such evidence is available, lactate-informed exercise should be described as a testable framework for precision exercise psychiatry, not as a ready clinical standard.

## 7 Conclusion and future perspectives

Exercise psychiatry is evolving from a behavioral framework toward a more mechanistic and phenotype-sensitive discipline. The central question is no longer only whether exercise can reduce depressive symptoms, but also which biological signals are generated by exercise, which patients are most likely to respond, and which symptom dimensions are most strongly affected. This review proposes that exercise-induced lactate should be considered as a dynamic immunometabolic signal rather than only as a metabolic byproduct or energetic substrate. During appropriately dosed exercise, lactate may rise as a transient and recoverable pulse. HCAR1/GPR81 provides one receptor-mediated route through which immune, vascular, and brain-associated cells may detect this pulse. The lactate-HCAR1 framework extends, but does not replace, established models involving BDNF, monoamines, HPA-axis regulation, hippocampal plasticity, myokines, mitochondrial adaptation, autonomic regulation, and behavioral reinforcement. Its main contribution is to place skeletal muscle metabolism, bone marrow myeloid output, peripheral inflammation, vascular and brain-border interfaces, and glial responses into a cross-organ hypothesis relevant to inflammation-related depressive symptom dimensions. These include fatigue, anhedonia, diminished motivation, psychomotor slowing, cognitive inefficiency, and sleep-wake disruption. The evidence supporting this model is uneven. Exercise-induced lactate kinetics are well established in human physiology, and HCAR1-dependent lactate signaling is supported by receptor biology and selected preclinical studies. Evidence linking exercise-induced lactate-HCAR1 signaling to bone marrow myeloid remodeling and neuroinflammation in depression-relevant models is promising but remains primarily preclinical. Direct evidence that this complete pathway operates in patients with depression is currently limited. Therefore, lactate-HCAR1 should be described as a candidate immunometabolic mechanism and not as an established mediator of exercise’s antidepressant effects. The translational implication is a research agenda rather than an immediate clinical prescription. Future studies should test whether lactate kinetics, including rise, peak, time above threshold, area under the curve, clearance, and recovery profile, predict changes in inflammatory biomarkers, immune-cell phenotypes, reward-related or neuroinflammatory readouts, and symptom dimensions. They should also account for age, sex, fitness, metabolic status, medication use, sleep, diet, anxiety sensitivity, panic vulnerability, and baseline inflammatory burden. Comparisons with conventional heart-rate-, VO_2_-, and perceived-exertion-based prescriptions will be necessary. If validated, the lactate-HCAR1 model may help transform exercise from a broadly recommended lifestyle intervention into a more measurable, adaptable, and phenotype-informed intervention for inflammation-related depression. At present, however, its value lies in providing a testable framework for future mechanistic and translational studies.
